# Supplementary material for: Experimental warming accelerates positive soil priming in a temperate grassland ecosystem
Source: Nat Commun. 2024 Feb 8;15:1178. doi: 10.1038/s41467-024-45277-0 (PMC10853207; doi:10.1038/s41467-024-45277-0)
Supplement: Supplementary file 1 — Supplementary Information [file 41467_2024_45277_MOESM1_ESM.pdf]

Supplementary Information for

Experimental warming accelerates positive soil priming in a temperate grassland ecosystem

Xuanyu Tao[1,2,#], Zhifeng Yang[1,2,#], Jiajie Feng[1,2,#], Siyang Jian[1,2,#], Yunfeng Yang[3*], Colin T. Bates[1,2], Gangsheng Wang[4], Xue Guo[1,2,3], Daliang Ning[1,2], Megan L. Kempher[1,2], Xiao Jun A. Liu[1,2], Yang Ouyang[1,2], Shun Han[1,2], Linwei Wu[1,2], Yufei Zeng[3], Jialiang Kuang[1,2], Ya Zhang[1,2], Xishu Zhou[1,2], Zheng Shi[1,2], Wei Qin[1,2], Jianjun Wang[5], Mary K. Firestone[6,7], James M. Tiedje[8], and Jizhong Zhou[1,2,7,9,10*]

[1]Institute for Environmental Genomics, University of Oklahoma, Norman, OK 73019, USA;

[2]School of Biological Sciences, University of Oklahoma, Norman, OK 73019, USA;

[3]State Key Joint Laboratory of Environment Simulation and Pollution Control, School of Environment, Tsinghua University, Beijing 100084, China;

[4]Institute for Water-Carbon Cycles and Carbon Neutrality, and State Key Laboratory of Water Resources Engineering and Management, Wuhan University, Wuhan 430072, China

[5]State Key Laboratory of Lake Science and Environment, Nanjing Institute of Geography and Limnology, Chinese Academic of Sciences, Nanjing 210008, China

[6]Department of Environmental Science, Policy, and Management, University of California, Berkeley, Berkeley, California, CA 94720, USA

[7]Earth and Environmental Sciences, Lawrence Berkeley National Laboratory, Berkeley, CA 94720, USA.

[8]Center for Microbial Ecology, Michigan State University, East Lansing, MI 48824, USA

[9]School of Civil Engineering and Environmental Sciences, University of Oklahoma, Norman, OK 73019, USA

[10]School of Computer Sciences, University of Oklahoma, Norman, OK 73019, USA

[#]These authors contributed equally to this work

[*]Corresponding author: Yunfeng Yang, yangyf@tsinghua.edu.cn; Jizhong Zhou, jzhou@ou.edu

Supplementary Note 1. Long-term warming enhanced the positive priming effect

By analyzing $^{13}CO_2$, we can differentiate CO_2 derived from the added litter from that derived from native soil organic C. In the control samples, the 7-day cumulative litter-derived CO_2 amounted to 182.7 ± 16.4 μg C/g soil, and the native soil-derived CO_2 (i.e., native soil respiration) reached 143.3 ± 8.9 μg C/g soil, substantially exceeding basal soil respiration (i.e., soil respiration without litter addition, 22.2 ± 2.1 μg C/g soil) (Fig. 2b & Supplementary Fig. 2). In the warmed samples, basal soil respiration (24.8 ± 4.0 μg C/g soil) was similar to that of the control samples, while the litter-derived CO_2 rose to 208.0 ± 10.4 μg C/g soil ($p < 0.01$, permutation ANOVA) and native soil respiration rose to 160.4 ± 9.3 μg C/g soil ($p < 0.05$, permutation ANOVA). Consequently, microbial respiration in the warmed samples was significantly higher ($p < 0.01$, permutation ANOVA) than in the control samples, with an increase of 14.2% ± 12.8% (Fig. 2b & Supplementary Fig. 2).

The SIP experiment requires a short-term incubation to minimize cross-feeding[1,2]. Accordingly, we set the incubation period at one week. However, this one-week incubation may not capture the effects of more recalcitrant carbon sources, such as lignin, on priming. Moreover, while previous studies have used similar or even higher amounts of complex C to assess priming effects[3-5], it remains unclear whether smaller C additions would yield comparable results, especially for the priming effect in response to warming. Therefore, to account for the possibility of continued carbon processing beyond the initial seven-day incubation period, we established an additional 63-day incubation experiment. In this extended experiment, we aimed to assess the priming effect of both warming and control samples with reduced straw addition 0.33 g of ^{13}C-straw in 5 g of soil (equivalent to 3 mg C /g dry soil) over a longer timeframe. As expected, the

general patterns of the priming effects were consistent between the 63-day incubation and the 7-day incubation experiments (Fig. 2b, Supplementary Fig. 2 & Fig. 3), but the magnitudes of positive priming effects were different in the two experiments due to the changes in quantity of oat straw during the experimental periods. The overall priming effect in the 63-day incubation experiment was significantly higher ($p < 0.050$, permutation ANOVA) for the soil samples under warming than control, with an increase of 27.8% ± 8.1% (Supplementary Fig. 3).

Supplementary Note 2. Warming amplified active bacterial abundance and stimulated potential C assimilation

A total of 7,945 amplicon sequence variants (ASVs), also known as phylotypes, of 16S rRNA gene sequences were identified across all samples and fractions. Of these, only 147 ASVs were identified as active C decomposers. Therefore, we calculated bacterial abundance by the sum of each ASV abundance, following the standard protocol in the original qSIP study[6]. Warming increased active bacterial abundance by 81% ± 17% ($p < 0.001$, permutation ANOVA) and total bacterial abundance by 44% ± 12% ($p < 0.001$, permutation ANOVA, Fig. 2c). Since microorganisms vary substantially in their 16S rRNA gene copy numbers, ranging from 1 to 15[7], the abundance of microorganisms with high copy numbers of the 16S rRNA gene may be overestimated, while those with low copy numbers may be underestimated. We addressed this issue by adjusting bacterial abundance by copy numbers in the reference genomes. Still, we found that warming significantly increased both active and total bacterial abundance (Supplementary Fig. 5).

Supplementary Note 3. Warming restructured microbial community structure

The proportion of active ASVs relative to the total abundance is 65% ± 3% for the warming group and 53% ± 8% for the control group. Well-known C decomposers, such as *Burkholderia*, *Sphingomonas,* and *Bacillus*[8-10], were among the 147 ASVs identified as active C decomposers (Fig. 2d). More than half of the active ASVs belonged to the phylum *Proteobacteria*, followed by 31 *Actinobacteria*, 11 *Bacteroidetes,* and 10 *Firmicutes* ASVs. We also detected 29 unclassified active genera, suggesting that our understanding of soil C-decomposers in temperate grasslands is quite limited. Similar to a previous finding that experimental warming in tundra soils increased the phylogenetic α-diversity of active bacterial community[8], warming treatment in this study increased the phylogenetic α-diversity of active bacterial community (Supplementary Fig. 8a). Based on a mixed-effects meta-regression model analysis[11], soil temperature was the only significant factor affecting the α-phylogenetic diversity of active bacterial community (Supplementary Fig. 8b). In sharp contrast, warming did not change the phylogenetic α-diversity of total bacterial community (p = 0.58, permutation ANOVA, Supplementary Fig. 8c). Interestingly, warming did not affect the taxonomic diversity (richness) of the active bacterial community (p = 0.67), but increased that in the total bacterial community ($p < 0.05$, permutation ANOVA) (Supplementary Fig. 9). Fifty-six ASVs were active only in warmed samples, most of which were from *α-Proteobacteria* (36%), *Bacillales* of *Firmicutes* (16%), *Actinobacteria* (16%), and *Bacteroidetes* (14%) (Fig. 2d). Consistently, the community assembly analysis indicated that environmental selection at our warming site primarily affected the *Bacillales* order (Supplementary Fig. 7), which contains many efficient C decomposers[10]. Active *Bacillales* ASVs contained high gene copy numbers of the 16S rRNA gene (Fig. 2d), potentially enabling rapid growth responses to environmental changes[12-14]. Primed C was strongly positively correlated with

the abundance of ASV 2 ($r = 0.72$, $p < 0.05$), belonging to *Bacillales*. The ASV2 was very abundant, with accounting for 5.01% ± 3.08%. Therefore, *Bacillales* could be important C-decomposing responders strongly affected by warming, consistent with our recent study[15]. Forty eight out of 56 active ASVs detected only in warmed samples by qSIP were also detected by annual measurements of total bacterial community during 2010–2016[15]. The mean relative abundance of these ASVs, almost exclusively belonging to *α-Proteobacteria*, *Bacillales*, *Actinobacteria*, and *Bacteroidetes*, increased by 27–205% under warming (Fig. 2f), verifying our SIP experimental results. In contrast, warming did not affect the mean relative abundance of 53 ASVs active only in the control samples or 38 ASVs found in both warmed and control samples (Supplementary Fig. 6). Most of the ASVs active only in control samples belonged to *Proteobacteria*, especially *β*- and *r-Proteobacteria* (Supplementary Fig. 6). Our results demonstrated a substantial compositional change induced by warming, which could affect soil C decomposition. Similarly, experimental warming shifted active ligninolytic communities from *β-Proteobacteria* to *α-Proteobacteria* in tundra soils[8]. Finally, despite the biomass ratio suggesting higher bacterial biomass, fungi undeniably play a pivotal role in litter decomposition. While our study focused primarily on bacterial contributions, it is worth noting that a holistic understanding of soil processes would benefit from a balanced exploration of both bacterial and fungal roles.

Supplementary Note 4. Warming enhanced the priming effect by regulating both active and inactive bacterial communities

A current conceptual model has been proposed to explain the microbial mechanisms underlying the soil priming effect. According to this model, priming arises as fast growing *r*-strategists consume fresh C inputs and indirectly stimulate slower growing *k*-strategist that then consume

additional native soil C [16-19]. These ecological strategies have been further posited to align with phylogenetic groups. However, using the qSIP technique (both ^{13}C-glucose and ^{18}O-H_2O), Morrissey et al. found that most taxa whose growth was increased by glucose addition ended up consuming a mix of glucose and native SOC[20], suggesting a direct stimulation of activity within individual taxa. Consequently, to gain deeper insights into the microbial mechanisms underlying this phenomenon, we further examined the relationships between taxa in both active (species responsive to fresh carbon inputs such as straw) and 'inactive' (species unresponsive to fresh carbon inputs such as straw) communities, and soil priming to understand how they are interconnected. At the family taxonomic level[21], there was a significant positive correlation between primed C and six families (e.g., *Planococcaceae, Rhizobiaceae*, *Sphingomonadaceae*, etc., Supplementary Table 3) within the active bacterial communities, as well as six families (e.g., *Acanthopleuribacteraceae*, *Clostridiales_Incertae Sedis III*, *Chloroflexaceae*, etc., Supplementary Table 4) within the inactive bacterial communities. Notably, among these highly correlated families, the average 16S rRNA gene copy number in active communities (6.7 ± 1.1) was significantly higher ($p < 0.001$, permutation ANOVA) than that in inactive communities (2.4 ± 0.3). Warming did not affect the average 16S rRNA gene copy number in both active and inactive communities ($p > 0.050$, permutation ANOVA). However, warming did increase the abundance of these highly correlated families in active communities ($p < 0.050$, permutation ANOVA) (Supplementary Fig. 12). At the ASV taxonomic level, two ASVs (ASV_2 and ASV_45) from active communities and four ASVs (ASV_162, ASV_289, ASV_1112, and ASV_1624) from inactive communities showed a significant correlation with primed C (Supplementary Table 5). Likewise, the average 16S rRNA gene copy number (9.7 ± 0.3) for those ASVs in active communities was significantly higher than that (2.7 ± 1.3) for the ASVs from inactive communities.

Warming increased the abundance of these highly correlated ASVs in both active (p = 0.090, permutation ANOVA) and inactive communities ($p < 0.050$, permutation ANOVA) (Supplementary Fig. 13). Also, warming did not affect the average 16S rRNA gene copy number in both active and inactive communities ($p > 0.050$, permutation ANOVA).

The disparities in the 16S rRNA gene copy numbers between correlated active and inactive taxa suggest that the correlated organisms in the active communities were more likely to be copiotrophic or *r*-strategists (indicated by relatively high 16S rRNA gene copy numbers), whereas those in the inactive communities were more likely to be oligotrophic or *k*-strategists (indicated by relatively low 16S rRNA gene copy numbers). Since warming increased the abundances of both correlated families and ASVs, this suggests that the enhanced abundance, rather than the rRNA gene copy numbers, could be one of the factors contributing to the warming-induced priming effect. This finding is consistent with the results of our PLS model analysis (Figure 4a).

The correlation between primed C and specific families (or ASVs) in both active and inactive communities implies that both groups contribute to the consumption of additional native SOC (priming effect). This observation aligns more closely with Morrissey et al.'s study[20], which found that most taxa, whose growth were stimulated by fresh carbon addition, ended up consuming a mix of fresh carbon and native SOC. Similarly, our results contradict the hypothesis that *r*-strategist organisms consume labile C, indirectly stimulating *k*-strategist microorganisms to consume additional SOC. In fact, marginal or significant correlations were observed between primed C and active functional gene groups involved in degrading nearly all carbon compounds targeted by GeoChip, including starch, hemicellulose, cellulose, chitin, phospholipids, and vanillin/lignin (Supplementary Table 6). This suggests that many taxa, whose growth is stimulated by the straw

addition, also participate in the soil priming effect. While we identified several families or ASVs from the inactive to fresh C community that are highly correlated with the soil priming effect, the absence of $^{18}O\text{-}H_2O$ qSIP data means that we cannot confirm that these organisms are actively consuming the additional native SOC. This remains an area for further investigation in future studies.

Supplementary Note 5. Relative importance of parameters for simulated variables and generalization of models

During the development of MEND, we have used the Multi-Objective Parameter Sensitivity Analysis (MOPSA) method to identify key parameters[22,23]. MOPSA calculates a sensitivity index (SI) by comparing parameter distributions that yield acceptable and unacceptable objective function values. The most influential parameters for simulating microbial biomass and enzyme pools were Initial active fraction of microbes (*r0*), maximum specific growth rate (V_g), a ratio ($\alpha=V_{mt}/(V_g+V_{mt})$) relating specific maintenance rate (V_{mt}) to V_g (α) , half-saturation constant for microbial assimilation of the substrate (K_D), and growth yield at reference temperature (Y_g). Among these, α had the greatest impact on simulated CO_2 flux. When considering all pools and CO_2 eflux, Vmt, enzyme turnover rate (*rE*), *Yg*, enzyme production rate (*pEP*), and half-saturation constant (*K*) for P_O were also crucial for prediction accuracy. For the current MEND application in field simulations, we conducted additional sensitivity analyses using the MOPSA method for a greater number of calibrated parameters and targeted variables, as shown in Supplementary Fig. 16a. The results indicated that the simulated variables are sensitive to different combinations of parameters; for instance, soil organic matter (SOM) was most sensitive to changes in Qmax (maximum sorption capacity), while microbial biomass carbon (MBC) was

mostly sensitive to changes in Yg. By averaging the sensitivity index (SI) ranks for targeted variables, Yg, Vg, and KD emerged as the top three influential parameters, whereas fD and gD were the least influential among the 14 parameters selected during the calibration of field-MEND. As there might be a risk of overfitting with the inclusion of more parameters in the calibration, we tested the generalization of the MEND model to unused data alongside an increase in calibrated parameters. The dataset for the Control treatment was divided into a training set (first 3/4 data) for model calibration, and a test set (subsequent 1/4 data) for evaluating the model's generalization to unused data. Using the calibrated models with increased calibrated model parameters, we calculated the training and test errors ($1-R^2$, unexplained variation) in predicting R_h with training and test sets. If the training error decreases but the test error increases with an increasing number of model parameters, the model could be overly complex, fitting too closely to the training set and failing to accurately predict unused data, which would suggest overfitting due to an excessive number of parameters[24]. Our results showed that the test error exhibited a decreasing trend within the range of parameter numbers, indicating that the model is not overfitted due to the increasing number of parameters (see Supplementary Fig. 16b). However, it is noteworthy that the test error was consistently higher than the training error across models of varying complexity. This discrepancy may be indicative of potential overfitting, influenced by other factors such as the limited or imbalanced data and model structure. Addressing the data limitation could involve extending the experimental period or increasing the frequency of data collection, while resolving the model structure limitation might require enhanced mechanistic understanding of biogeochemical processes and subsequent model development. Nonetheless, our results suggested that an increase in the number of parameters did not lead to the overfitting of the model.

Increasing model complexity can potentially heighten parameter uncertainty when additional data is not used to estimate the parameters. Therefore, we assessed the parameter uncertainty for the aforementioned calibration of the training set across different levels of model complexity. As expected, parameter uncertainty increased with the number of parameters until it reached around 11 (refer to Supplementary Fig. 16c). However, contrary to our expectations, the curve then began to decline, suggesting that incorporating more parameters could actually decrease parameter uncertainty. This counterintuitive result implies that parameters with low sensitivity indices may still be important for capturing certain data variations. Selecting a simpler model with fewer parameters and using the data which are not sufficiently informative for those parameters might not significantly impact the goodness-of-fit, even if parameter uncertainty is high. Yet, this high parameter uncertainty may not pose a problem for calibration data but could potentially compromise the model's generalization when applied to other datasets. In summary, the influences of model complexity on uncertainty initially increase as the number of parameters grows but subsequently begins to taper off.

Supplementary Table 1 | Environmental variables in 2016

Environmental factors	Control	Warming	*p*-value[a]
Soil carbon content (%)	0.798 ± 0.051[b]	0.888 ± 0.060	0.359
Soil nitrogen content (%)	0.088 ± 0.005	0.096 ± 0.006	0.384
**Soil nitrate (mg kg^{-1})**	**2.25 ± 0.90**	**11.45 ± 3.53**	**2.5×10^{-5}**
Soil ammonia (mg kg^{-1})	3.51 ± 0.62	2.87 ± 0.51	0.427
Soil pH	7.30 ± 0.13	7.19 ± 0.18	0.666
**Soil temperature at 7.5 cm depth (°C)[c]**	**17.15 ± 0.19**	**18.88 ± 0.37**	**0.029**
Soil moisture (% v/v)	11.75 ± 1.17	10.02 ± 0.98	0.365
Soil moisture when sampled (% v/v)	24.48 ± 2.60	22.64 ± 1.12	0.4
Aboveground plant biomass (g m^{-2})	204.9 ± 60.6	107.2 ± 22.7	0.239

[a]The Significance is determined by using one-sided permutation ANOVA.

[b]Values shown in this table are average ± standard error of n = 4 biological replicates.

[c]Bold font represents a significant difference ($p < 0.050$) between warmed and control samples.

Supplementary Table 2 | Potential predictors (independent variables, X) for each factor (dependent variable, Y) in partial least squares (PLS) analysis.

No.	Dependent variable (Y)	Potential predictors (independent variables, X, in PLS)[a]
**1**	Soil total carbon	Soil temperature, Soil moisture, Soil pH, Soil total nitrogen, Aboveground plant biomass, Bacterial phylogenetic diversity, Bacterial beta-diversity (PCs), Active bacterial abundance, Relevant functional gene families, Respiration, Priming C, C assimilation rate.
**2**	Soil total nitrogen	Soil temperature, Soil moisture, Soil pH, Soil total carbon, Aboveground plant biomass, Bacterial phylogenetic diversity, Bacterial beta-diversity (PCs), Active bacterial abundance, Relevant functional gene families, Priming C, C assimilation rate.
**3**	Mineral nitrogen	Soil temperature, Soil moisture, Soil pH, Soil total nitrogen, Aboveground plant biomass, Active bacterial abundance, Relevant functional gene families[b], Priming C, C assimilation rate.
**4**	Aboveground plant biomass	Soil temperature, Soil moisture, Soil pH, Soil total nitrogen, Mineral nitrogen, Total C/N ratio, Bacterial phylogenetic diversity, Bacterial beta-diversity (PCs), Active bacterial abundance, Relevant functional gene families.
**5**	Bacterial phylogenetic diversity	Soil temperature, Soil moisture, Soil pH, Soil total carbon, Soil total nitrogen, Mineral nitrogen, Aboveground plant biomass, Total C/N ratio, The ratio of aboveground plant biomass to mineral nitrogen, Bacterial beta-diversity (PCs), Active bacterial abundance, Respiration, Priming C, C assimilation rate.
**6**	Bacterial beta-diversity	Soil temperature, Soil moisture, Soil pH, Soil total carbon, Soil total

		nitrogen, Mineral nitrogen, Aboveground plant biomass, Total C/N ratio, The ratio of aboveground plant biomass to mineral nitrogen, Bacterial phylogenetic diversity, Active bacterial abundance, Respiration, Priming C, C assimilation rate.
**7**	Active bacterial abundance	Soil temperature, Soil moisture, Soil pH, Soil total carbon, Soil total nitrogen, Mineral nitrogen, Aboveground plant biomass, Total C/N ratio, The ratio of aboveground plant biomass to mineral nitrogen, Bacterial phylogenetic diversity, Bacterial beta-diversity (PCs).
**8**	Each of the relevant functional gene families	Soil temperature, Soil moisture, Soil pH, Soil total carbon, Soil total nitrogen, Mineral nitrogen, Aboveground plant biomass, Total C/N ratio, The ratio of aboveground plant biomass to mineral nitrogen, Bacterial phylogenetic diversity, Bacterial beta-diversity (PCs), Active bacterial abundance.
**9**	Respiration	Soil temperature, Soil moisture, Soil pH, Soil total carbon, Soil total nitrogen, Mineral nitrogen, Aboveground plant biomass, Total C/N ratio, The ratio of aboveground plant biomass to mineral nitrogen, Bacterial phylogenetic diversity, Bacterial beta-diversity (PCs), Active bacterial abundance, Relevant functional gene families, Respiration, C assimilation rate.
**10**	Priming C	Soil temperature, Soil moisture, Soil pH, Soil total carbon, Soil total nitrogen, Mineral nitrogen, Aboveground plant biomass, Total C/N ratio, The ratio of aboveground plant biomass to mineral nitrogen, Bacterial phylogenetic diversity, Bacterial beta-diversity (PCs), Active bacterial abundance, Relevant functional gene

		families, Respiration, C assimilation rate.
**11**	C assimilation rate	Soil temperature, Soil moisture, Soil pH, Soil total carbon, Soil total nitrogen, Mineral nitrogen, Aboveground plant biomass, Total C/N ratio, The ratio of aboveground plant biomass to mineral nitrogen, Bacterial phylogenetic diversity, Bacterial beta-diversity (PCs), Active bacterial abundance, Relevant functional gene families, Respiration, Priming C.

[a]All the listed predictors were tested, and the optimum model was based on forward selection (see *Methods* for details). Soil temperature, moisture, and pH were not considered as dependent variables because their key determinants are out of the scope of this study.

[b]Functional gene families include C-degradation gene associated with decomposition of starch, hemicellulose, pectin, cellulose, phospholipids, chitin, lignin, vanillin_lignin.

Supplementary Table 3 | Mantel analysis between families from active communities and primed C.

**Family[a]**	***r*[b]**	***p*[c]**
Flavobacteriaceae	0.604	0.030
Nocardioidaceae	0.485	0.037
Planococcaceae	0.545	0.040
Rhizobiaceae	0.468	0.026
Rhodospirillaceae	0.323	0.039
Sphingomonadaceae	0.362	0.050

[a]Only Families with significant correlation ($p < 0.050$) are presented.
[b]Mantel analysis is based on Pearson correlation coefficient between Bray-Curtis dissimiliarity and difference of primed C.
[c]The Significance is determined by using one-sided Mantel test. No adjustments were made for multiple comparisons.

Supplementary Table 4 | Mantel analysis of the structures of families from inactive communities and primed C.

Family[a]	r[b]	*p*[c]
Acanthopleuribacteraceae	0.292	0.036
Chloroflexaceae	0.361	0.009
Clostridiales_Incertae Sedis III	0.453	0.022
Paenibacillaceae 2	0.328	0.017
Thermoleophilaceae	0.255	0.047
Xanthobacteraceae	0.527	0.027

[a]Only Families with significant correlation ($p < 0.050$) are presented.

[b]Mantel analysis is based on Pearson correlation coefficient between Bray-Curtis dissimiliarity and difference of primed C.

[c]The Significance is determined by using one-sided Mantel test. No adjustments were made for multiple comparisons.

Supplementary Table 5 | Pearson correlation between the abundances of ASVs from active or inactive communities and primed C.

ASV[a]	Genus	*r*	*p*[b]	Group
ASV_2	*Bhargavaea*	0.717	0.045	Active
ASV_45	*Sphingomonas*	0.836	0.010	Active
ASV_1112	*Paenisporosarcina*	0.719	0.044	Inactive
ASV_162	*Nitrososphaera*	0.717	0.045	Inactive
ASV_1624	*Belnapia*	0.691	0.049	Inactive
ASV_289	*Gemmatimonas*	0.760	0.029	Inactive

[a]Only ASVs with significant correlation ($p < 0.050$) are presented.

[b]The Significance is determined by using two-sided Pearson correlation test. No adjustments were made for multiple comparisons.

Supplementary Table 6 | Pearson correlation between abundances of each functional gene group involved in C degradation and Primed C.

Carbon groups	***r***	***p***[b]
Cellulose	0.405	0.090
Chitin	0.441	0.072
**Hemicellulose**[a]	**0.679**	**0.012**
Pectin	0.355	0.119
Phospholipids	0.471	0.060
Starch	0.439	0.074
**Vanillin/Lignin**	**0.767**	**0.004**

[a]Bold font represents a significant difference ($p < 0.050$) between warmed and control samples.
[b]The Significance is determined by using two-sided Pearson correlation test. No adjustments were made for multiple comparisons.

Supplementary Table 7 | Best-fit microbial parameter values and their 90% percentiles based on qSIP data assimilation

Parameter	Description	Best-fit value	90% percentile
p_{EP}	enzyme production rate	0.13	(0.009–0.023)
V_{g}	maximum specific growth rate	0.05	(0.04–0.05)
α	the ratio ($\alpha=V_{\mathrm{mt}}/(V_{\mathrm{g}}+V_{\mathrm{mt}})$) relating specific maintenance rate (V_{mt}) to V_g	0.13	(0.10–0.19)
K_D	half-saturation constant for microbial assimilation of the substrate	0.5	(0.42–0.5)
Y_g	intrinsic C use efficiency at reference temperature	0.28	(0.25–0.32)

Supplementary Table 8 | Governing equation for each soil C pool in the MEND model

C pool variation	Equation	
Particulate organic carbon (POC) decomposed by oxidative enzymes (P_{O})	$\frac{dP_{\mathrm{O}}}{dt} = I_{PO} + (1 - g_D) \cdot F_{12} - F_1$	(S1)
POC pool decomposed by hydrolytic enzymes (P_{H})	$\frac{dP_{\mathrm{H}}}{dt} = I_{PH} - F_2$	(S2)
Mineral-associated organic carbon (MOC, *M*)	$\frac{dM}{dt} = (1 - f_D) \cdot (F_1 + F_2) - F_3$	(S3)
Adsorbed DOC (QOC, *Q*)	$\frac{dQ}{dt} = F_4 - F_5$	(S4)
Dissolved organic carbon (DOC, *D*)	$\frac{dD}{dt} = I_D + f_D \cdot (F_1 + F_2) + g_D \cdot F_{12} + F_3 + \left(F_{14,EPO} + F_{14,EPH} + F_{14,EM}\right) - F_6 - (F_4 - F_5)$	(S5)
MBA	$\frac{dBA}{dt} = F_6 - (F_7 - F_8) - (F_9 + F_{10}) - F_{12} - \left(F_{13,EPO} + F_{13,EPH} + F_{13,EM}\right)$	(S6)
MBD	$\frac{dBD}{dt} = (F_7 - F_8) - F_{11}$	(S7)
Enzymes for P_{O} ($\mathrm{EP_O}$)	$\frac{dEP_1}{dt} = F_{13,EPO} - F_{14,EPH}$	(S8)
Enzymes for P_{H} ($\mathrm{EP_H}$)	$\frac{dEP_2}{dt} = F_{13,EPO} - F_{14,EPH}$	(S9)
Enzymes for M (EM)	$\frac{dEM}{dt} = F_{13,EM} - F_{14,EM}$	(S10)
Respiration(CO_2)	$\frac{dCO_2}{dt} = (F_9 + F_{10}) + F_{11}$	(S11)
Carbon balance	$\frac{d}{dt}(P_{\mathrm{O}} + P_{\mathrm{H}} + M + Q + D + BA + BD + EP_{\mathrm{O}} + EP_{\mathrm{H}} + EM) = I_{P1} + I_{P2} + I_D - (F_9 + F_{10} + F_{11})$	(S12)

Supplementary Table 9 | Component fluxes in the MEND model

Flux description	Equation	
Particulate organic carbon (POC) pool 1 (P_O) decomposition (F_1)	$F_1 = V_{PO} \cdot EP_O \cdot P_O/(K_{PO} + P_O)$	(S13)
POC pool 2 (P_H) decomposition	$F_2 = V_{PH} \cdot EP_H \cdot P_H/(K_{PH} + P_H)$	(S14)
Mineral-associated organic carbon (MOC, *M*) decomposition	$F_3 = V_M \cdot EM \cdot M/(K_M + M)$	(S15)
Adsorption (F_4) and desorption (F_5) between dissolved organic carbon (DOC, *D*) and adsorbed DOC (QOC, *Q*)	$F_4 = k_{ads} \cdot (1 - Q/Q_{max}) \cdot D$<br>$F_5 = k_{des} \cdot (Q/Q_{max})$	(S16)<br>(S17)
DOC (*D*) uptake by microbes	$F_6 = \frac{1}{Y_g} \cdot (V_g + V_{mt}) \frac{D \cdot BA}{K_D + D}$	(S18)
Dormancy (F_7) and reactivation (F_8) between active (MBA) and dormant (MBD) microbial biomass (*BA* and *BD*)	$F_7 = [1 - D/(K_D + D)] \cdot V_{mt} \cdot BA$<br>$F_8 = D/(K_D + D) \cdot V_{mt} \cdot BD$	(S19)<br>(S20)
MBA (*BA*) growth respiration (F_9) and maintenance respiration (F_{10})	$F_9 = (\frac{1}{Y_g} - 1) \frac{V_g \cdot D \cdot BA}{K_D + D}$<br>$F_{10} = (\frac{1}{Y_g} - 1) \frac{V_{mt} \cdot D \cdot BA}{K_D + D}$	(S21)<br>(S22)
MBD (*BD*) maintenance respiration	$F_{11} = \beta \cdot V_{mt} \cdot BD$	(S23)
MBA (*BA*) mortality	$F_{12} = \gamma \cdot V_{mt} \cdot BA$	(S24)
Synthesis of enzymes for P_1 (EP_O, $F_{13,EPO}$), enzymes for P_H (EP_H, $F_{13,EPH}$), and enzymes for *M* (*EM*, , $F_{13,EM}$)	$F_{13,EP1} = P_O/(P_O + P_H) \cdot P_{EP} \cdot V_{mt} \cdot BA$<br>$F_{13,EPH} = P_H/(P_O + P_H) \cdot P_{EP} \cdot V_{mt} \cdot BA$<br>$F_{13,EM} = P_{EM} \cdot V_{mt} \cdot BA$	(S25)
Turnover of enzymes (EP_1, EP_2, *EM*)	$F_{14,EPO} = r_E \cdot EP_O$<br>$F_{14,EPH} = r_E \cdot EP_H$<br>$F_{14,EM} = r_E \cdot EM$	(S26)

Supplementary Table 10| Response functions of soil pH, temperature, and moisture in MEND model.

Function description	Equation	Eq#
Reaction rate (v) at a specific soil water potential (ψ), soil temperature (T), and soil pH (pH)	$v = v_{ref} \cdot f(\psi) \cdot f(T) \cdot f(pH)$	(S27)
Response function of soil pH	$f(pH) = \exp [-\left[\frac{pH - pH_{opt}}{pH_{sen}}\right]$	(S28)
Temperature sensitivity of carbon use efficiency (Yg)	$Y_g(T) = Y_g(T_{ref}) - k_{Yg}(T - T_{ref})$	(S29)
Arrhenius equation or Q10 method to simulate the response of other parameters to changes in temperature	$f(T) = \exp\left[-\frac{Ea}{R}\left(\frac{1}{T} - \frac{1}{T_{ref}}\right)\right]$	(S30)
	$f(T) = Q_{10}^{\frac{T - T_{ref}}{10}}$	(S31)
	$Q_{10} = \exp\left[\frac{Ea}{R \cdot T_{ref}} \cdot \frac{10}{T}\right]$	(S32)
Soil moisture response function for SOM decomposition by oxidative enzymes	$f_{lig}(\psi) = \begin{cases} 0, \ \psi \le -10^{2.5} \\ 0.625 - 0.25 \times log_{10}(-\psi), \ -10^{2.5} < \psi \le -10^{1.5} \\ 1, \ -10^{1.5} < \psi \le -10^{-2.5} \\ \frac{[2.5 \ + \ 0.4 \ \times \ log10(-\psi)]}{1.5}, -10^{2.5} < \psi \le -10^{-4} \\ 0.6, \ \psi > -10^{-4} \end{cases}$	(S33)
Soil moisture response function for SOM decomposition by hydrolytic enzymes	$f_{cel} = \begin{cases} 0, \psi \le \psi_{min} \\ 1 - \left[\frac{ln\left(\frac{\psi}{\psi_{fc}}\right)}{ln\left(\frac{\psi}{\psi_{min}}\right)}\right]^b, \psi_{min} < \psi \le \psi_{fc} \\ 1, \psi > \psi_{fc} \end{cases}$	(S34)
Soil moisture response function for microbial mortality, dormancy & resuscitation	$f_{A2D}(\psi) = \frac{(-\psi)^{\omega}}{(-\psi)^{\omega} + (-\psi_{A2D})^{\omega}}$	(S35)
	$f_{D2A}(\psi) = \frac{(-\psi_{D2A})^{\omega}}{(-\psi)^{\omega} + (-\psi_{D2A})^{\omega}}$	(S36)

Supplementary Table 11 | MEND model parameters

ID	Parameter	Description	Prior range	Initial Parameter value	Units
1	LF_0	Initial fraction of POC_1 in POC	(0.1, 1.0)	0.3	—
2	r_0	Initial active fraction of microbes	(0.01, 1)	0.2	—
3	*fINP*	Scaling factor for litter input rate	(0.1,1)		
4	V_{PO}	Max specific decomposition rate for P_O	(0.1, 100)	75	mg C·mg^{-1} C·h^{-1}
5	V_{PH}	Max specific decomposition rate for P_H	(0.1, 100)	75	mg C·mg^{-1} C·h^{-1}
6	V_M	Max specific decomposition rate for *M*	(0.1, 100)	75	mg C·mg^{-1} C·h^{-1}
7	K_{PO}	Half-saturation constant (*K*) for P_O decomposition	(40, 100)	100	mg C·g^{-1} soil
8	K_{PH}	*K* for P_H decomposition	(1, 40)	6	mg C·g^{-1} soil
9	K_M	*K* for MOC decomposition	(100, 1000)	492	mg C·g^{-1} soil
10	Q_{max}	Max sorption capacity	(0.5, 5)	2.5	mg C·g^{-1} soil
11	K_{ba}	Binding affinity	(1, 16)	6	(mg C·g^{-1} soil)$^{-1}$
12	k_{des}	Desorption rate	(0.0001, 0.01)	0.006	mg C·g^{-1} soil·h^{-1}
13	r_E	Turnover rate of enzymes	(0.0001, 0.01)	0.01	mg C·mg^{-1} C·h^{-1}
14	p_{EP}	[$V_m \times p_{EP}$] is the production rate of *EP* (EP_1 + EP_2), V_m is the specific maintenance rate for active microbes	(0.0001, 0.05)	0.01	—
15	fp_{EM}	$fp_{EM} = p_{EM}/p_{EP}$, [$V_{mt} \times p_{EM}$] is the production rate of *EM*	(0.5, 3.0)	1	—
16	f_D	Fraction of decomposed POC allocated to DOC	(0.05, 1)	0.5	—
17	g_D	Fraction of dead microbes allocated to DOC	(0.01, 1)	0.5	—
18	V_g	Max specific growth rate	(0.001, 0.1)	0.05	mg C·mg^{-1} C·h^{-1}
19	α	$= V_{mt} /(V_g + V_{mt})$	(0.01, 0.5)	0.04	—
20	K_D	*K* for microbial uptake	(0.01, 0.5)	0.26	mg C·g^{-1} soil
21	Y_g	True growth yield at reference temperature (T_{ref})	(0.2, 0.6)	0.3	—
22	k_{Yg}	Temperature slope for Y_g	(0.001,0.016)	0.01	(°C)$^{-1}$
23	*Q10*	*Q10 for temperature response function*			
24	γ	Max microbial mortality rate = $V_m \times \gamma$	(0.1, 20)	1	—
25	β	Ratio of dormant maintenance rate to V_m	(0.0005,0.05)	0.001	—
26	ψ_{A2D}	Soil water potential (SWP) threshold for microbial dormancy	(−0.6, −0.2)	−0.4	MPa

27	τ	$\psi_{D2A} = \psi_{A2D} \times \tau$, ψ_{D2A} is the SWP threshold for microbial resuscitation	(0.1, 0.95)	0.25	—
28	ω	Exponential in SWP function for microbial dormancy or resuscitation	(1, 6)	4	—

Supplementary Table 12 | Objective functions used for different response variables in the MEND model parameterization

Response variables	Description	Objective function
Cumulative CO_2 efflux	Cumulative CO_2 calculated from laboratory-measured daily respiration rate	*MARE* between simulation and observation
R_h	*In situ* heterotrophic respiration	R^2 between simulated R_h and observed R_h
MBC	*In situ* reference MBC value = 2% × soil organic carbon content	*MARE* < 50% of reference MBC value
Active fraction	Active fraction = active MBC / total MBC	*MARE* between simulation and observation
EnzCo	Concentration (EnzC) of oxidative enzyme	*MARE* between simulated EnzC and expected EnzC Expected EnzC = Simulated EnzC at control × RR
EnzCh	Hydrolytic enzyme concentration	*MARE* between Simulated EnzC and Expected EnzC Expected EnzC = Simulated EnzC at control × RR

RR is the response ratio of gene abundance under warming to that under control. R^2 denotes the coefficient of determination, *MARE* is the mean absolute relative error, see Methods Eqs. 5-6 for details.

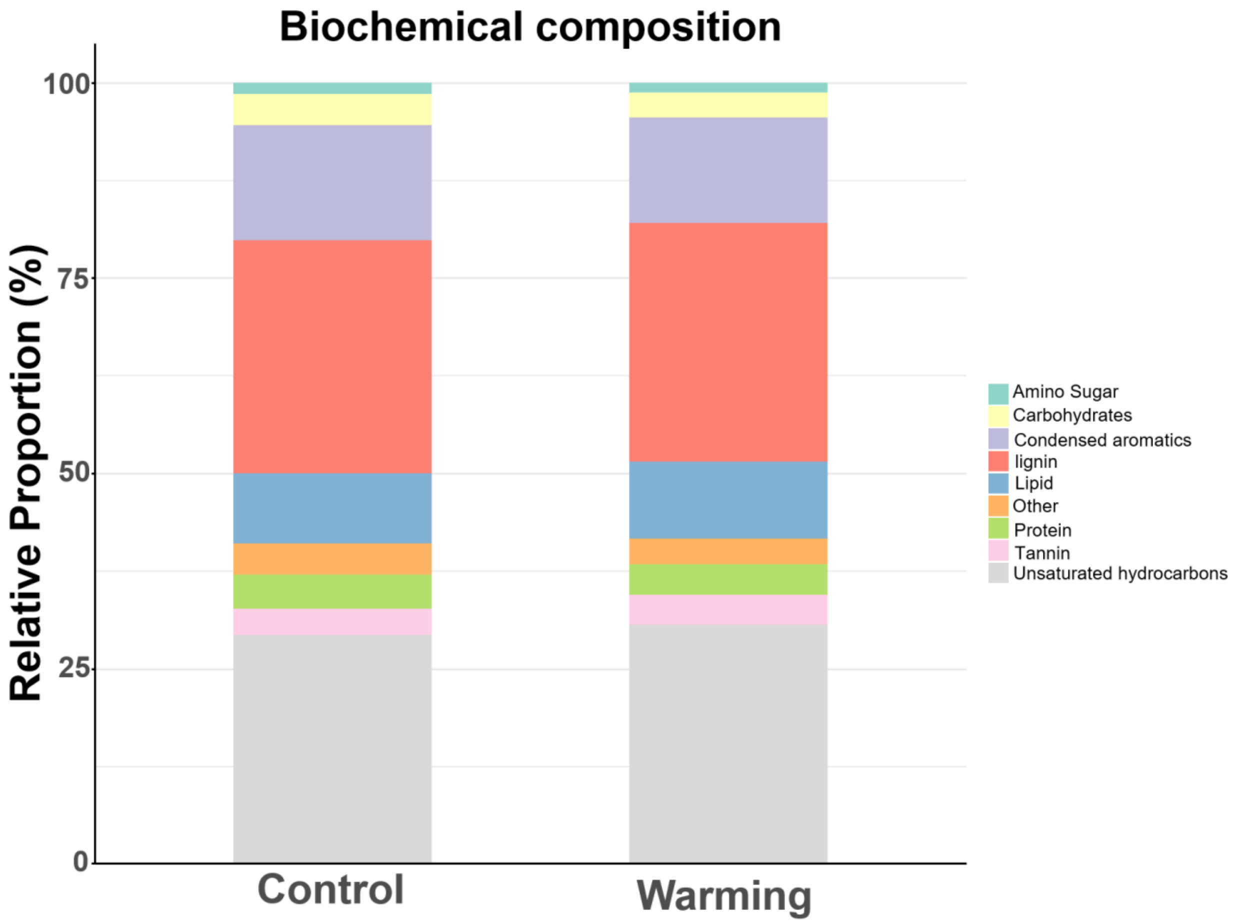

Supplementary Fig. 1 | Biochemical composition of DOM measured by FT-ICR MS. The relative proportion is the mean value of four replicates of warmed or control samples. The DOM colors are defined as follow: Amino Sugar (teal), Carbohydrates (yellow), Condensed aromatics (purple), Lignin (Red), Lipid (navy blue), Other (orange), Protein (light green), Tannin (pink), Unsaturated hydrocarbons (Grey). Source data are provided as a Source Data file.

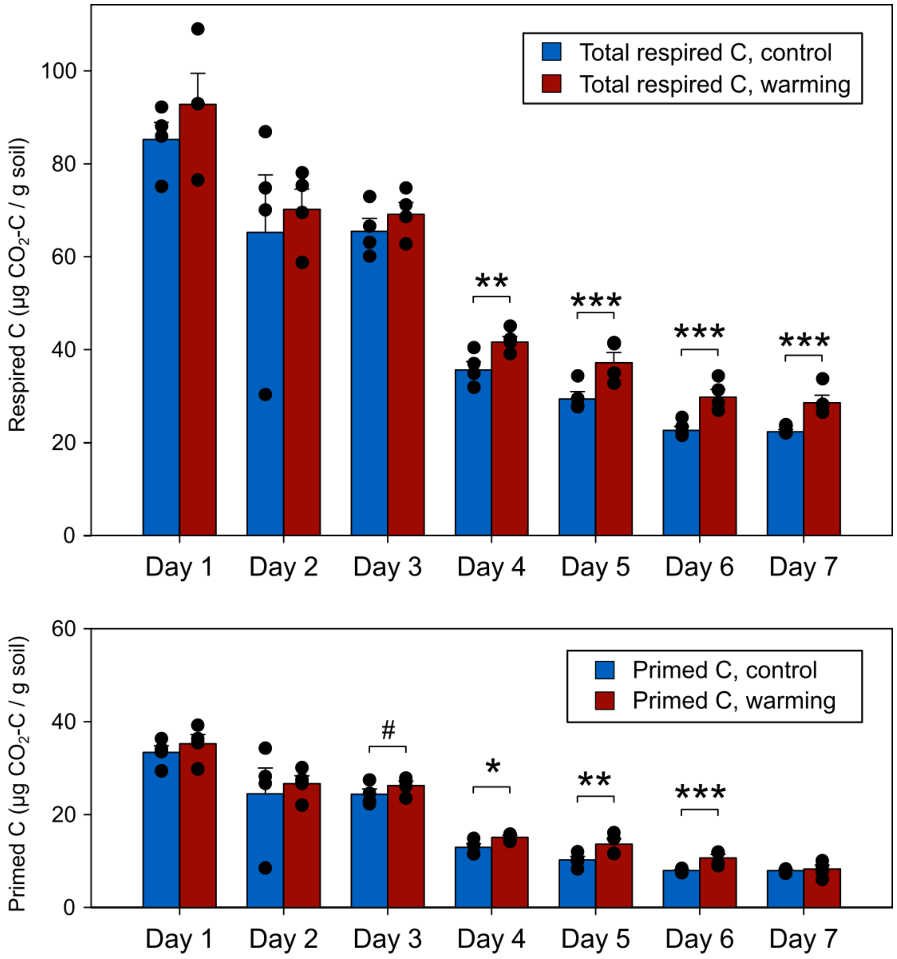

Supplementary Fig. 2 | Microbial respiration (a) and priming effect (b) on each day during the 7-day incubation with ^{13}C-labelled straw. The bars represent the average ± standard error of four biological replicates (n=4) of warmed (red) or control (blue) samples. Significance is denoted as follows: #, $p \leq 0.1$; *, $p \leq 0.05$; **, $p \leq 0.01$; and ***, $p \leq 0.001$ determined by using one-sided permutation ANOVA. No adjustments were made for multiple comparisons, and exact p-values are provided in the Source Data file. Source data are provided as a Source Data file.

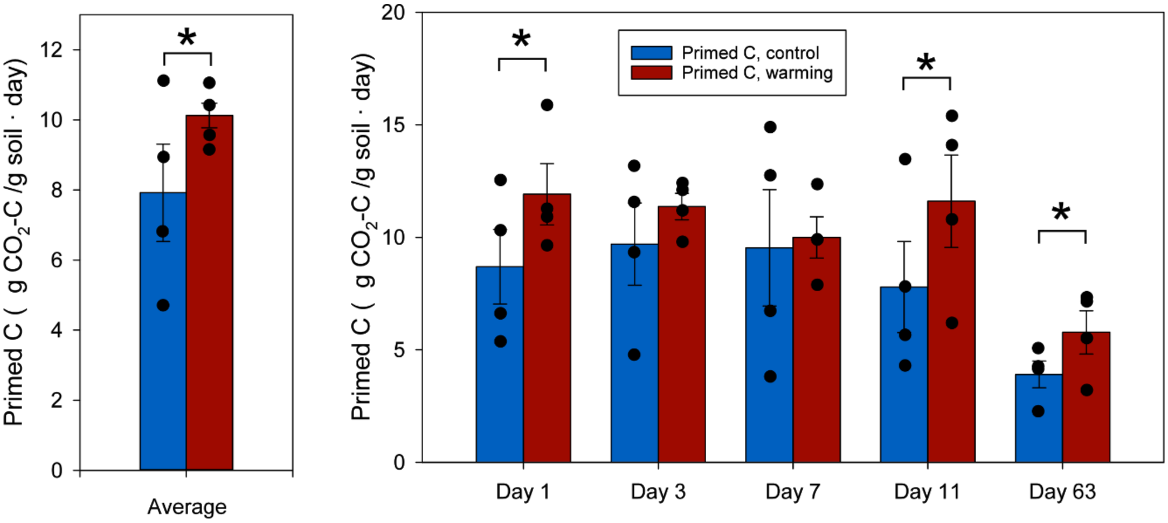

Supplementary Fig. 3 | The microbial priming effect during or 63-day incubation with ^{13}C-labelled straw. a, the average primed C amounts for both warming and control samples during 63-day incubation. **b**, the primed C amounts on each day during the 63-day incubation. These bars represent the mean ± standard error of four biological replicates (n=4) of warmed (red) or control (blue) samples. Significance is denoted as follows: *, $p \leq 0.05$, determined by using one-sided permutation ANOVA. No adjustments were made for multiple comparisons, and exact p-values are provided in the Source Data file. Source data are provided as a Source Data file.

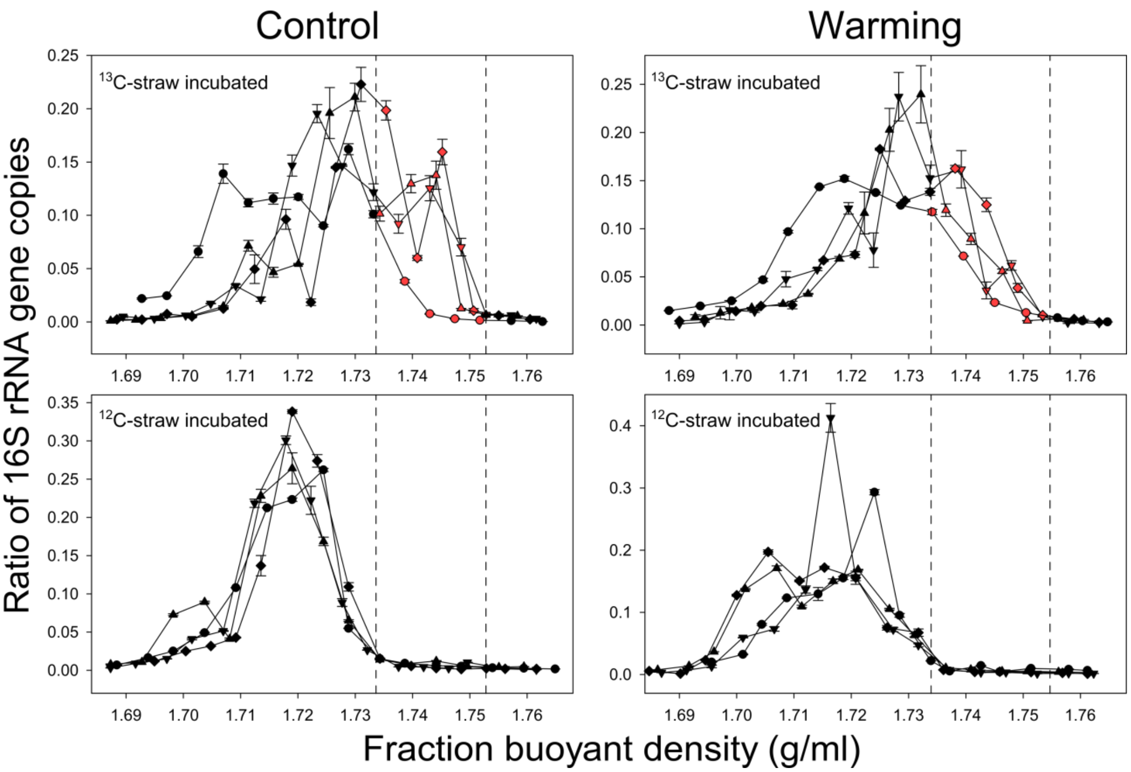

Supplementary Fig. 4 | Distribution of 16S rRNA gene abundances with buoyant density. In each pane, Up triangles, down triangles, diamonds, and circles represent fractions of biological replicate 1, 2, 3, and 4, respectively. Red symbols in the panes of ^{13}C-plant litter represent fractions of active bacterial community, in which the corresponding ^{12}C-plant-litter-incubated samples at the same densities were close to zero. The symbols represent the mean ± standard error of three technical replicates of qPCR. Source data are provided as a Source Data file.

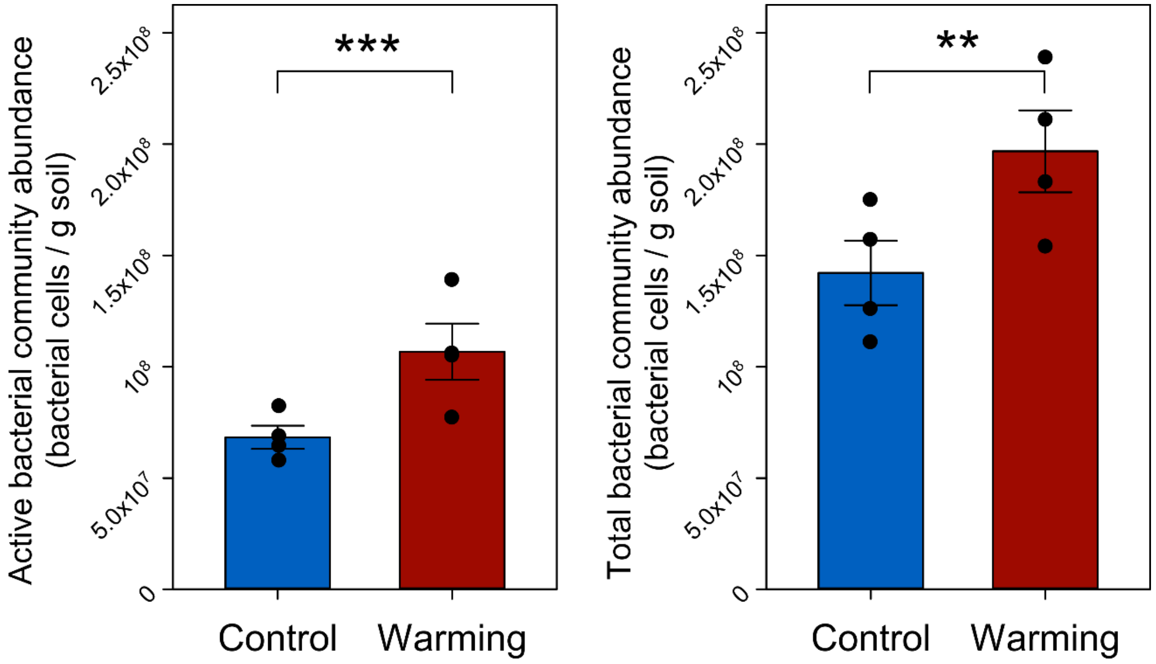

Supplementary Fig. 5 | Abundances of active and total bacterial community adjusted by 16S rRNA gene copy numbers. The columns represent the mean ± standard error of four biological replicates (n=4) of warmed (red) or control (blue) samples. Significance is denoted as follows: **, $p \leq 0.01$ and ***, $p \leq 0.001$, determined by using one-sided permutation ANOVA. Exact p-values are provided in the Source Data file. Source data are provided as a Source Data file.

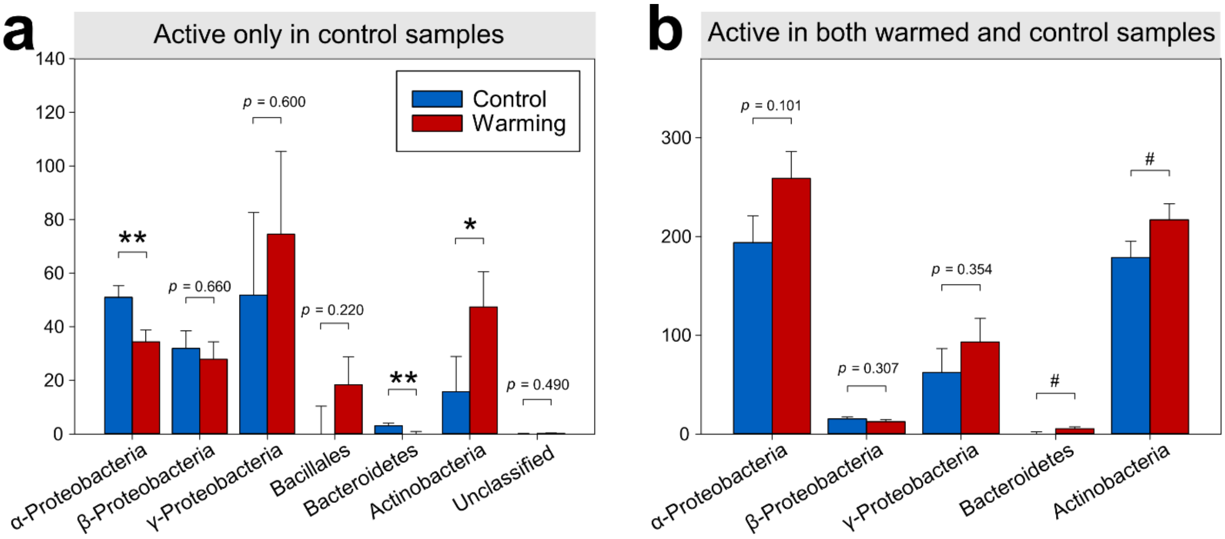

Supplementary Fig. 6 | Yearly means of relative abundance of active bacterial ASVs only in control samples (a) or shared in both warmed and control samples (b). The least-squares mean values were determined by the linear mixed-effects model. Each bar represents the mean ± standard error of 28 biological replicates (n=28) of *in situ* warming (red) or control (blue) samples over yearly repeated measures during 2010–2016. Significance is denoted as follows: #, $p \leq 0.1$; *, $p \leq 0.05$, and **, $p \leq 0.01$, determined by using ANOVA. No adjustments were made for multiple comparisons, and exact p-values are provided in the Source Data file. Source data are provided as a Source Data file.

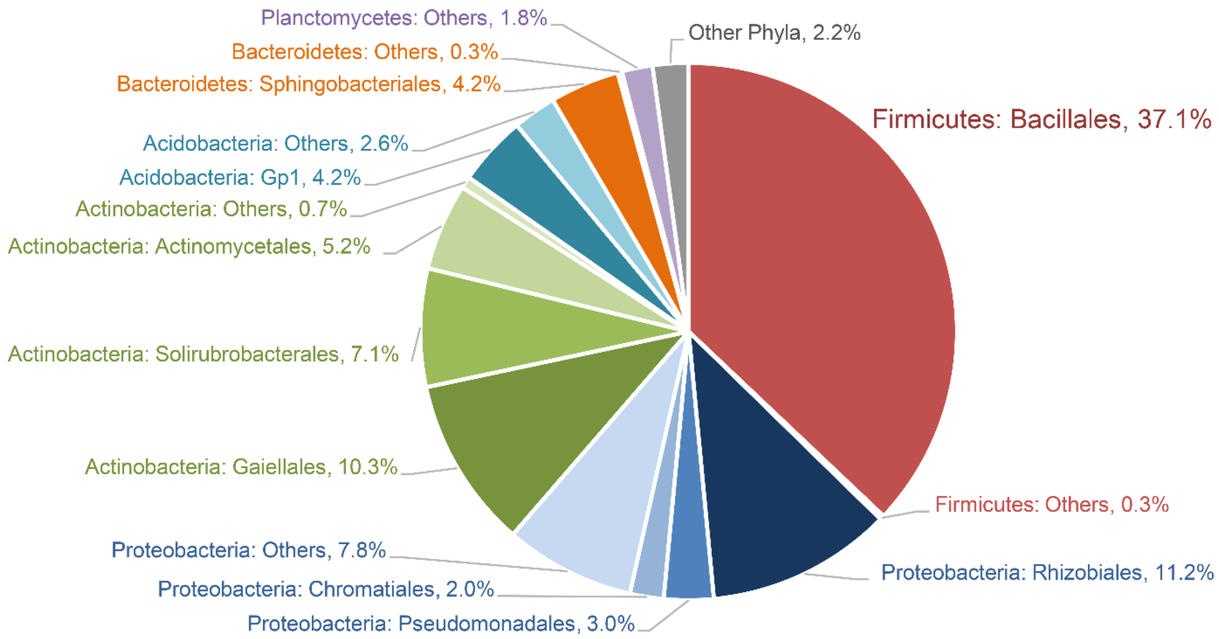

Supplementary Fig. 7 | Composition of phylogenetic groups affected by warming-enhanced selection. The analysis is based on 16S rRNA gene sequencing data of soil samples during 2010-2016. Source data are provided as a Source Data file.

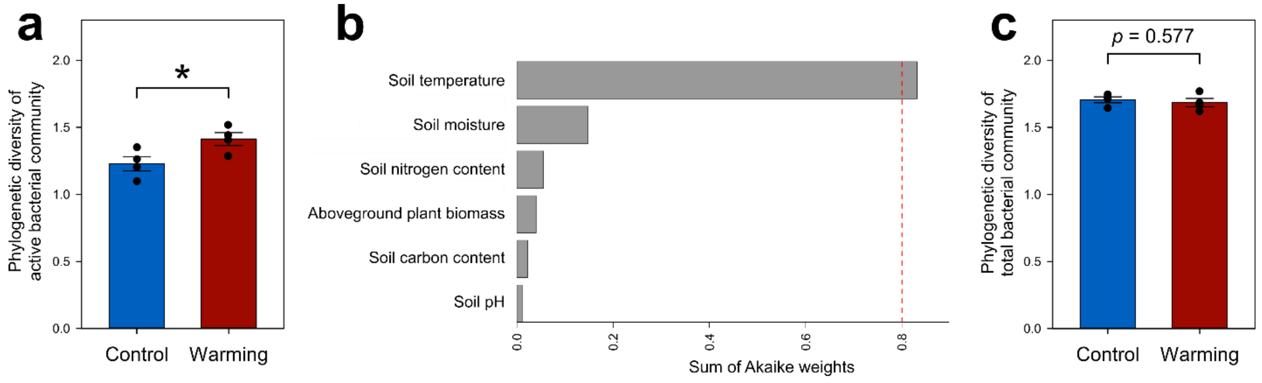

Supplementary Fig. 8 | Phylogenetic diversities and relative importance analysis after the 7-day qSIP incubation. a, The phylogenetic diversity of active bacterial community. The columns represent the mean ± standard error of four biological replicates (n=4) of warmed (red) or control (blue). Significance is denoted as follows: *, $p \leq 0.05$ determined by using two-sided ANOVA. Exact p-values are provided in the Source Data file. **b**, The relative importance of environmental factors in regulating phylogenetic diversity. The relative importance score was determined by a mixed-effects meta-regression model, and the red dash line indicates a threshold value of 0.8. **c**, The phylogenetic diversity of total bacterial community. The columns represent the mean ± standard error of four biological replicates (n=4) of warmed (red) or control (blue) samples. Significance is determined by using two-sided ANOVA. Source data are provided as a Source Data file.

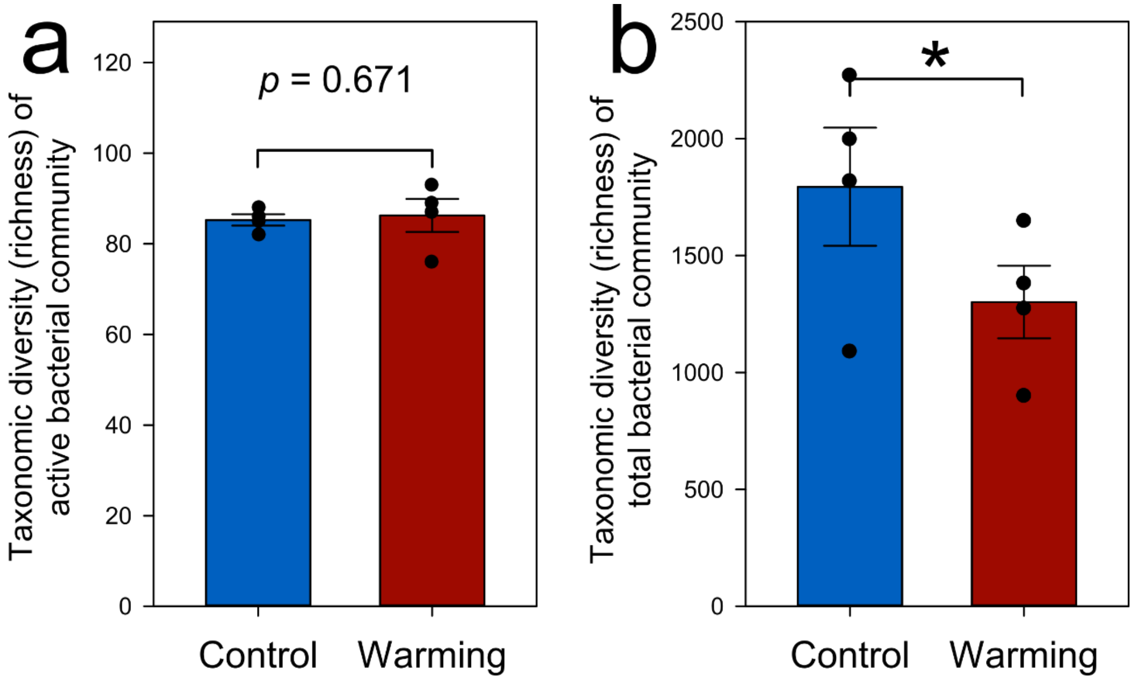

Supplementary Fig. 9 | Taxonomic diversity analysis after the 7-day qSIP incubation. a, The taxonomic diversity of active bacterial community. The columns represent the mean ± standard error of four biological replicates (n=4) of warmed (red) or control (blue) samples. Significance is determined by using one-sided ANOVA. **b**, The taxonomic diversity of total bacterial community. The columns represent the mean ± standard error of four biological replicates (n=4) of warmed or control samples. Significance is denoted as follows: *, $p \leq 0.05$ determined by using one-sided ANOVA. Exact p-values are provided in the Source Data file. Source data are provided as a Source Data file.

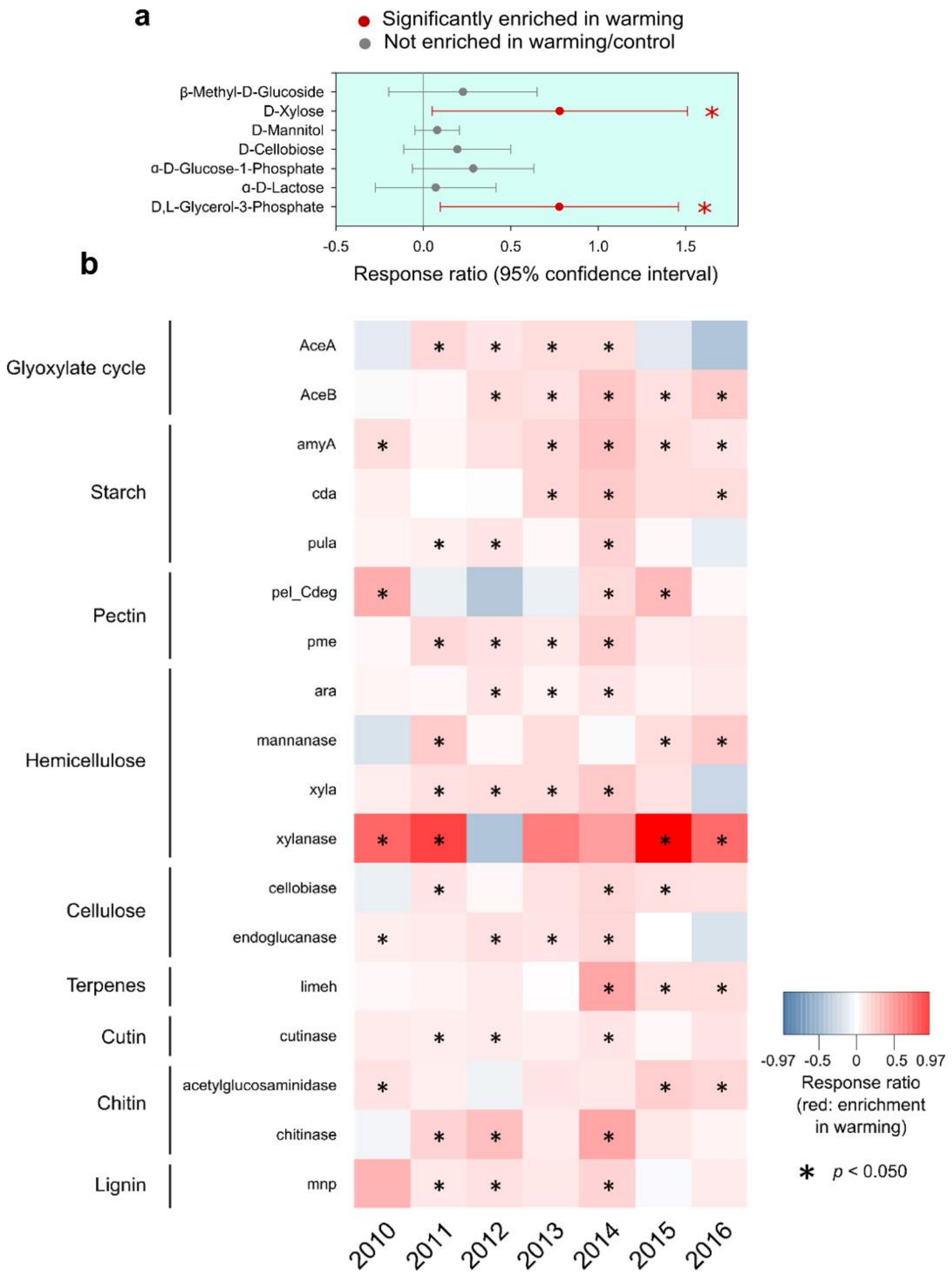
a
Significantly enriched in warming
Not enriched in warming/control
β-Methyl-D-Glucoside
D-Xylose
D-Mannitol
D-Cellobiose
α-D-Glucose-1-Phosphate
α-D-Lactose
D,L-Glycerol-3-Phosphate
-0.5
0.0
0.5
1.0
1.5
Response ratio (95% confidence interval)
b
Glyoxylate cycle
AceA
AceB
Starch
amyA
cda
pula
Pectin
pel_Cdeg
pme
Hemicellulose
ara
mannanase
xyla
xylanase
Cellulose
cellobiase
endoglucanase
Terpenes
limeh
Cutin
cutinase
Chitin
acetylglucosaminidase
chitinase
Lignin
mnp
2010
2011
2012
2013
2014
2015
2016
-0.97 -0.5 0 0.5 0.97
Response ratio
(red: enrichment
in warming)
* p < 0.050

Supplementary Fig. 10 | Warming stimulates the C-decomposing capacity in the field during 2010-2016. **a**, Response ratios of microbial carbohydrates utilization capacity determined by BIOLOG EcoPlates between the warming and control samples, which were measured before 7-day incubation. Red symbols represent significantly positive response ratios. Grey symbols represent non-significant response ratios. Each symbol represents the mean ± 95% CI of four biological replicates (n=4) of warmed or control samples. Significance is denoted as follows: *, $p \leq 0.05$, as determined by using the one-sided Response Ratio test[25]. **b**, Response ratios of key carbon cycling genes between the warming and control samples during 2010-2016. Red represents increased relative abundance in warming samples, while blue represents increased relative abundance in control samples. Significance is denoted as follows: *, $p \leq 0.05$, as determined by using the one-sided Response Ratio test[25] (95% CI). No adjustments were made for multiple comparisons. Source data are provided as a Source Data file.

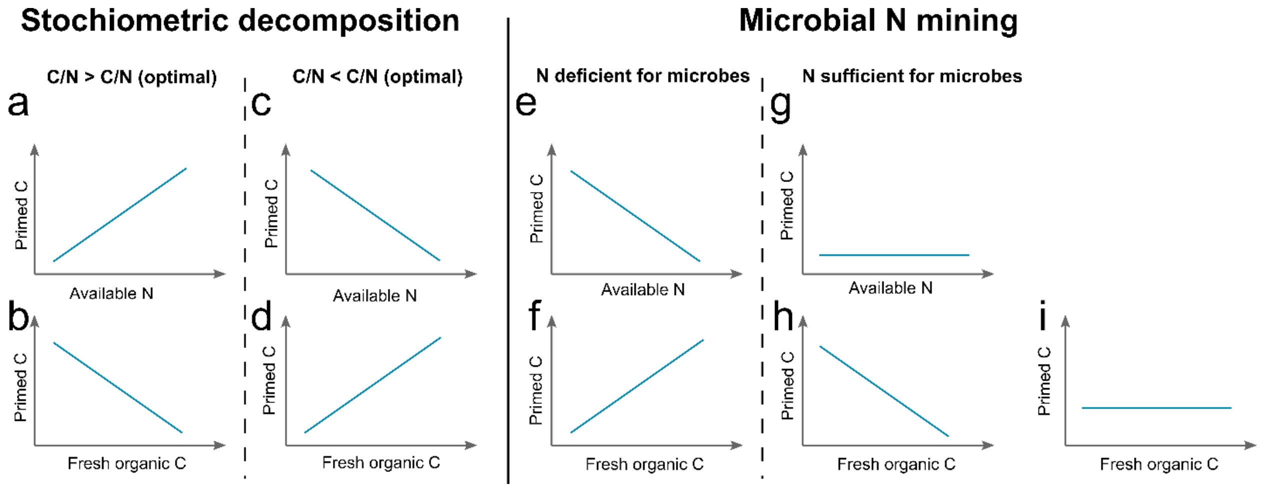

Supplementary Fig. 11 | Conceptual frameworks derived from the stoichiometric decomposition[26,27] and microbial N mining[28,29] hypotheses. The stoichiometric decomposition hypothesis proposes that microbial activity, including decomposition and respiration, is highest when substrate stoichiometry matches microbial demands. The microbial N mining hypothesis proposes that microorganisms use labile C as an energy source to decompose native SOC for additional N. **a**, if the C/N ratio exceeds the optimal C/N ratio of the microbial demand, with an increase in available N, the C/N ratio approaches the optimal stoichiometry, resulting in stronger microbial activity and consequently higher C decomposition and priming effect; **b,** With an increase of fresh C input, the C/N ratio deviates from the optimal stoichiometry, leading to a greater deficiency of C, weaker microbial activity, and lower C decomposition and priming effect. **c,** if the C/N ratio is lower than the optimal C/N ratio of the microbial demand , an increase in available N causes the C/N ratio to deviate further from the optimal C/N ratio, weaker microbial activity, and lower C decomposition and priming effect; **d,** with an increase of fresh C input, the C/N ratio approaches to the optimal stoichiometry, resulting in stronger microbial activity and consequently higher C decomposition and priming effect. **e**, The priming effect for 'N mining' mitigates N deficiency when N is deficient for microbes, but becomes less important as available N increases; **f**, An increase in fresh C input can result in a more pronounced priming effect due to both the relative N deficiency and the enhanced energy from labile C. **g**, If the N is sufficient for microbes, the priming effect becomes unnecessary and displays an N-independent pattern. **h**, when N is sufficient, but C is limited, microorganisms might resort to decomposing the native SOC for C,

leading to a decrease in the priming effect as fresh C input increases. **i**, When C isn't a limiting factor, the priming effect might not confer a noticeable advantage to microorganisms, hence showing no clear trend with changes in fresh C input.

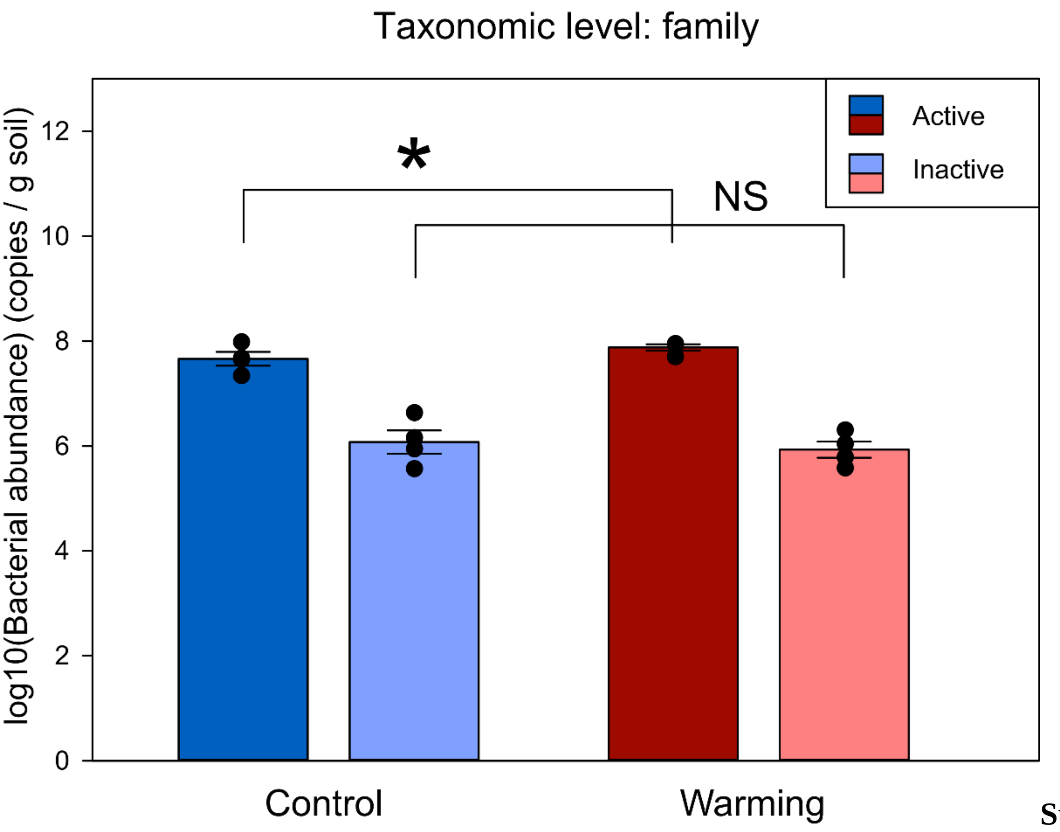

Supplementary

Supplementary Fig. 12| Abundance of families highly correlated with primed C in both active and inactive communities. The columns represent the mean ± standard error of four biological replicates (n=4) for the abundance of families that are highly correlated with primed C. Significance is indicated by *, $0.010 < p \leq 0.050$, determined by using one-sided permutation ANOVA. NS: not significant. Exact p-values are provided in the Source Data file. Source data are provided as a Source Data file.

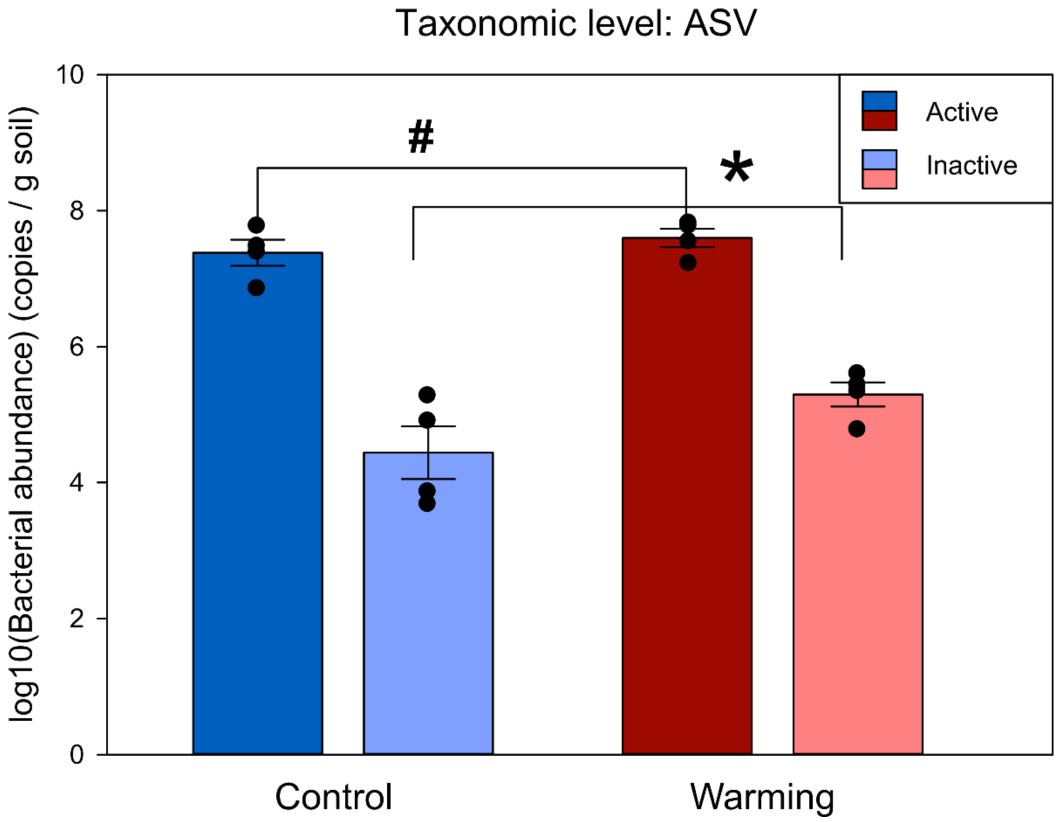

Supplementary Supplementary Fig. 13 | Abundance of ASVs highly correlated with primed C in both active and inactive communities. The columns represent the mean ± standard error of four biological replicates (n=4) for the abundance of ASVs that are highly correlated with primed C. Significance is indicated by #, $p \leq 0.10$; and *, $p \leq 0.05$, determined by using one-sided permutation ANOVA. Exact p-values are provided in the Source Data file. Source data are provided as a Source Data file.

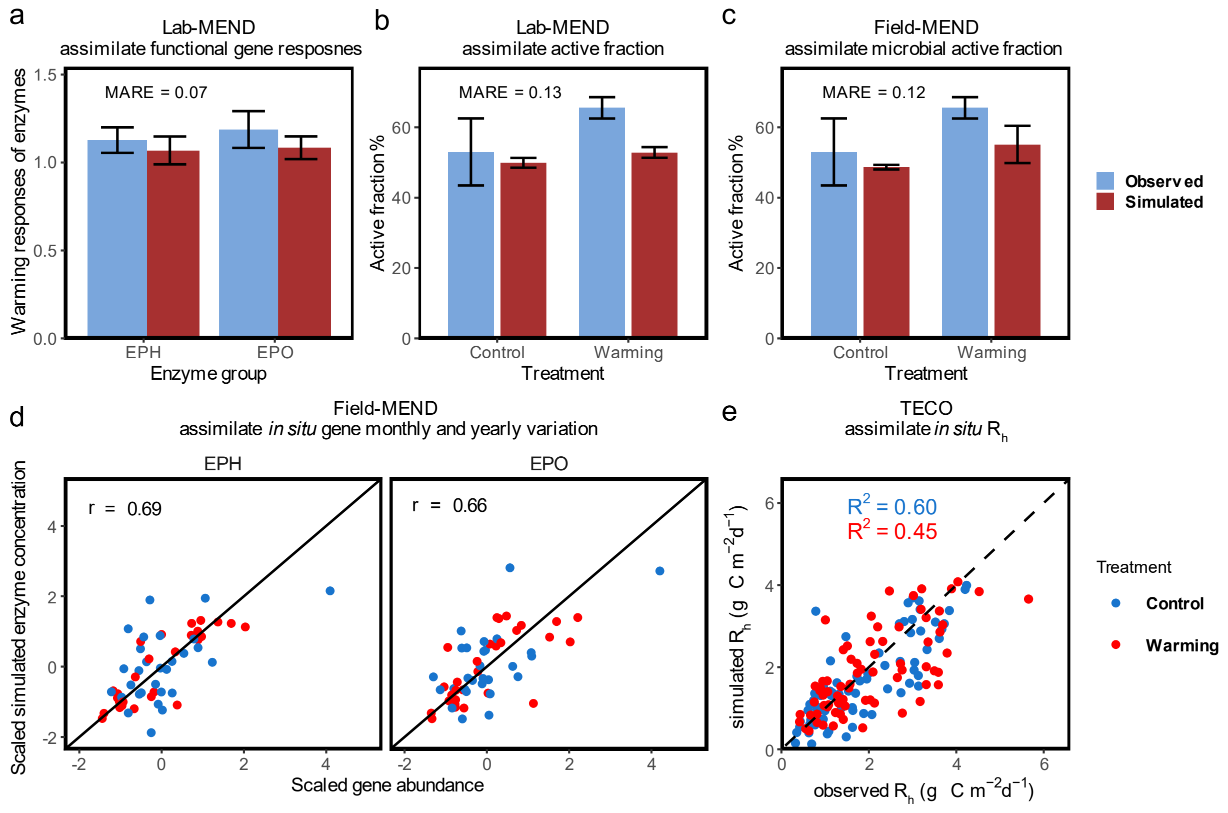

Supplementary Fig. 14| Calibration results of lab-MEND, field-MEND and TECO models. **a**, Lab-MEND simulated responses of EPO and EPH vs. observed responses microbial functional gene abundance. **b**, Lab-MEND simulated microbial active fractions vs. observed microbial active fractions. **c**, Field-MEND simulated microbial active fractions vs. observed microbial active fractions. **d**, Field-MEND simulated enzyme concentrations vs. observed in situ microbial functional gene abundance. **e**, TECO simulated in situ Rh vs. observed in situ Rh. EPO, EPH : oxidative enzymes and hydrolytic enzymes for degrading POC in MEND. As gene abundance and enzyme concentrations have different units, they cannot be compared directly. Alternatively, we compared their responses to warming (by dividing the values under warming with values under control) or temporal variation (by scaling it to a standard normal distribution), which would remove the unit differences. For figure a, b, c, data is shown as mean ± Standard Deviation (SD), n = 4 biological replicated for observed values, n = 24 hourly values for simulated values. MARE: mean absolute relative error. r: correlation coefficient. R^2: coefficient of determination. Source data are provided as a Source Data file.

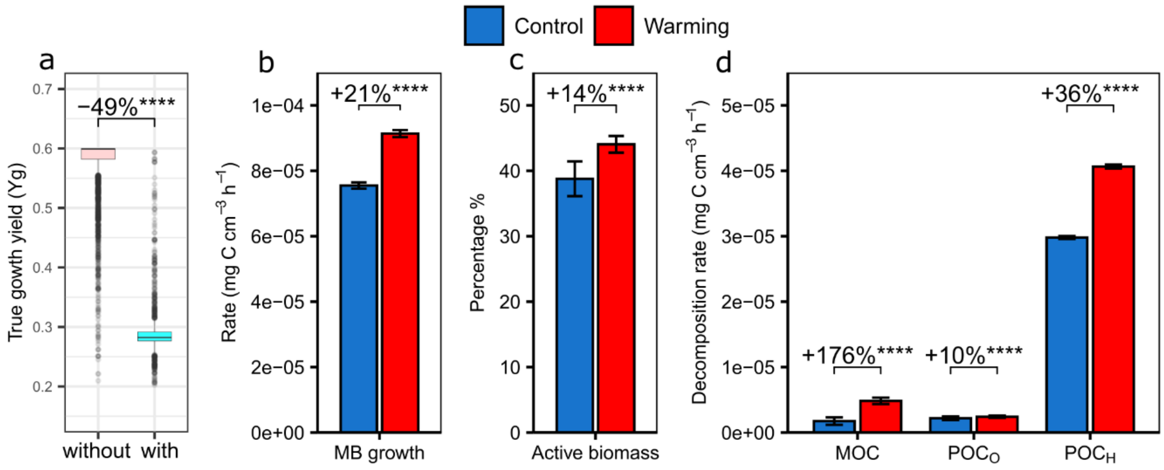

Supplementary Fig. 15 | MEND model simulations based on qSIP and field data. a, Comparison of the growth yield (Y_g) parameter uncertainty ranges without and with incorporation of active fraction data during calibration (n = 3692 accepted parameter values for without group, n = 1429 for with group determined by Critical Objective Function Index (COFI) method). **b,** Simulated microbial growth rate. **c,** Simulated active microbial fraction. **d,** Simulated decomposition rates of mineral-associated organic C (MOC), particulate organic C degraded by oxidative enzyme (POC_O) and particulate organic C degraded by hydrolytic enzyme (POC_H). The presented simulated microbial growth rate, active microbial fraction, and decomposition rates were the 2016 annual averages. The medians in boxplots are shown as a line, the boxes show the interquartile range (the 1st and 3rd quartiles), and the whiskers show the upper and lower extremes, determined to be equal to or less than 1.5 times the interquartile range against the 1st and 3rd quartiles. The error bar in figure b, c, and d represents mean ± SD (n = 11473 accepted calibrated models for Control and n = 8360 for Warming) of selected variable simulated by models using accepted parameter sets determined by COFI method. Significance is indicated by ****, $p < 0.0001$, determined by the two-sided Wilcoxon test. No adjustments were made for multiple comparisons, and exact p-values are provided in the Source Data file. Source data are provided as a Source Data file.

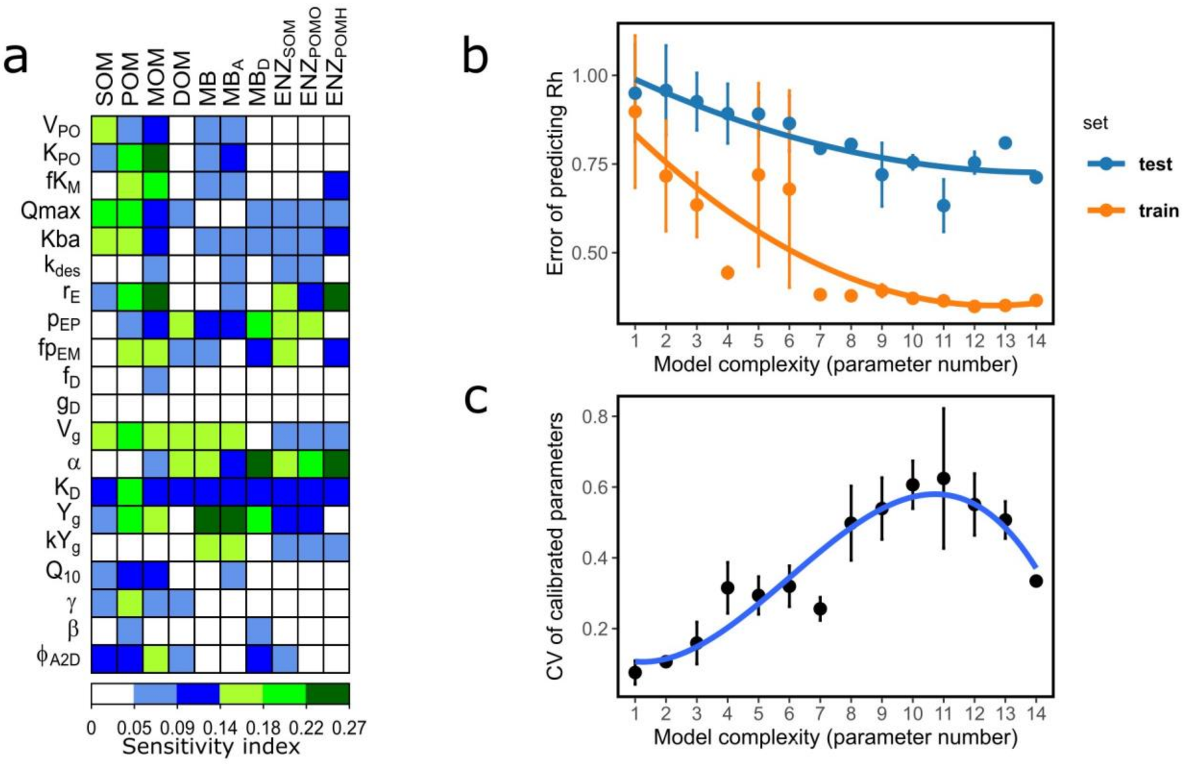

Supplementary Fig. 16 | MEND parameter sensitivity analysis and the impact of parameter number on model performance. a. The heatmap represents the sensitivity index for each parameter's effect on simulated variables in the MEND model. The sensitivity index is defined as the median of the discrepancies between acceptable and unacceptable parameter samples, as identified by the Multi-Objective Parameter Sensitivity Analysis (MOPSA) method. The terms ENZSOM, ENZPOMO, and ENZPOMH denote the sum of enzymes involved in C degradation and the enzyme pools EPO, EPH within MEND, respectively. The descriptions of variables and parameters are available in Supplementary Tables 8 and 11. **b.** This panel illustrates the influence of model complexity on training and test errors. The dataset, which includes variables such as Rh and gene abundance, was partitioned into a training set (the first 3/4 data) for model calibration and a test set (the subsequent 1/4 data) to evaluate model generalization. Model complexity is represented by calibrating different numbers of parameters, chosen based on their sensitivity index rankings from among 14 parameters in the field-MEND model. For each complexity level, three distinct parameter sets were evaluated. An increase in test error coupled with a decrease in training error, following the addition of parameters, could indicate overfitting to the training data[30]. Conversely, a trend of decreasing test error with an increasing number of calibrated parameters may suggest that the model is appropriately complex and not overfitted. Prediction errors are quantified as 1-R^2 when comparing simulated and observed Rh, with error bars indicating the standard error from the three parameter sets at each complexity level. **c.** The effect of model complexity on parameter uncertainty is quantified using the Coefficient of Variation (CV). The error bar in figure b and c represents mean ± Standard Error of Mean (SEM) (n = 3 combinations

of parameters). The CV for each complexity level is the average across all calibrated parameters. Source data are provided as a Source Data file.

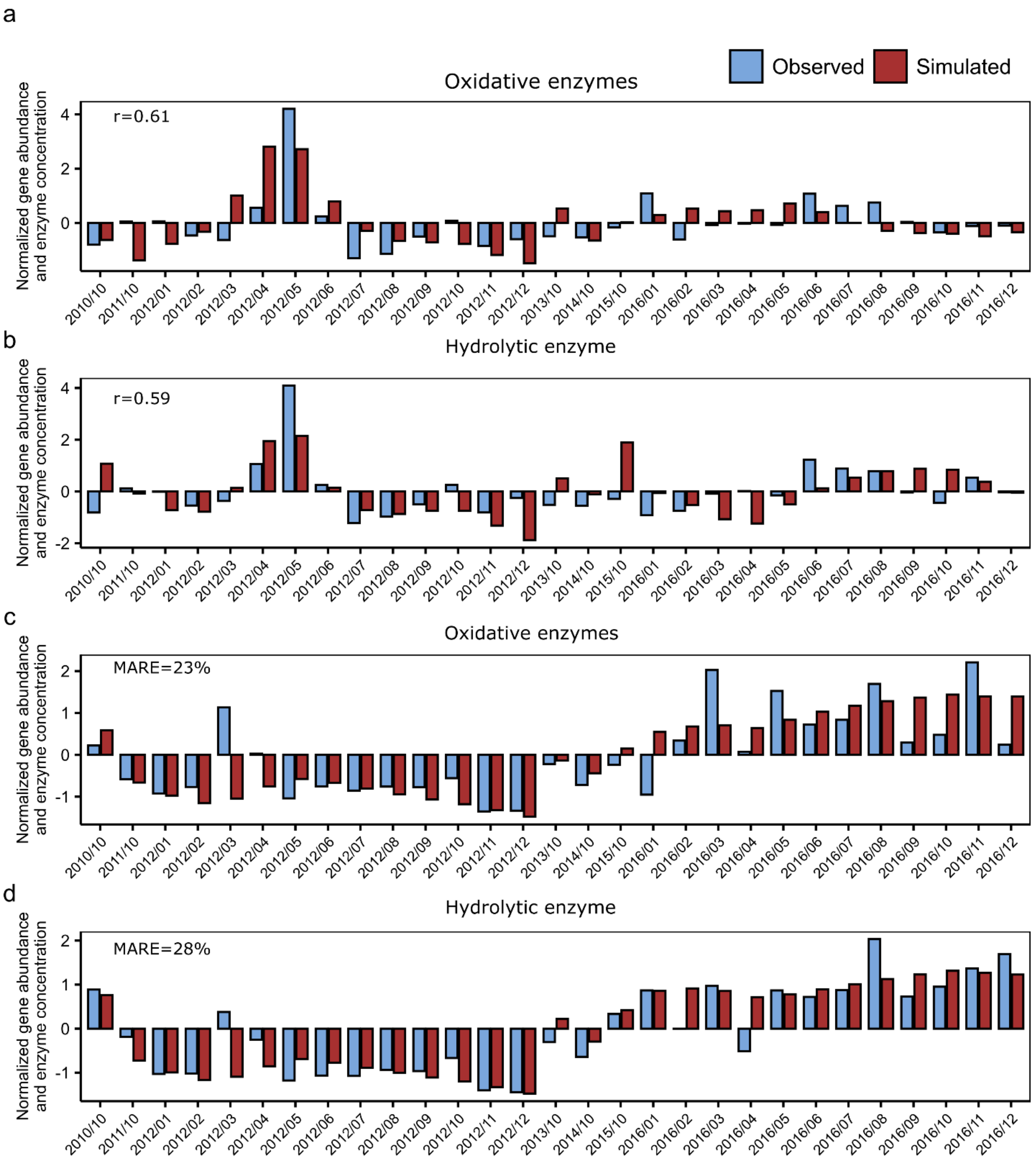

Supplementary Fig. 17 | Simulated monthly and yearly enzyme concentrations compared with observed GeoChip gene abundance. a, Simulated enzyme concentration vs. observed gene abundance for oxidative enzyme under control condition. **b**). Simulated enzyme concentration vs. observed gene abundance for hydrolytic enzyme under control condition. **c**). Simulated enzyme

concentration vs. observed gene abundance for oxidative enzyme under warming condition. **d**). Simulated enzyme concentration vs. observed gene abundance for hydrolytic enzyme under warming condition. To remove the unit and magnitude differences, we normalized the gene abundance and enzyme concentrations. The goodness-of-fit was either shown as correlation coefficient (r) to capture temporal variation or mean relative mean absolute relative error (MARE) to capture warming responses of enzymes (see supplementary Table 12 for the objective functions used in calibration). Source data are provided as a Source Data file.

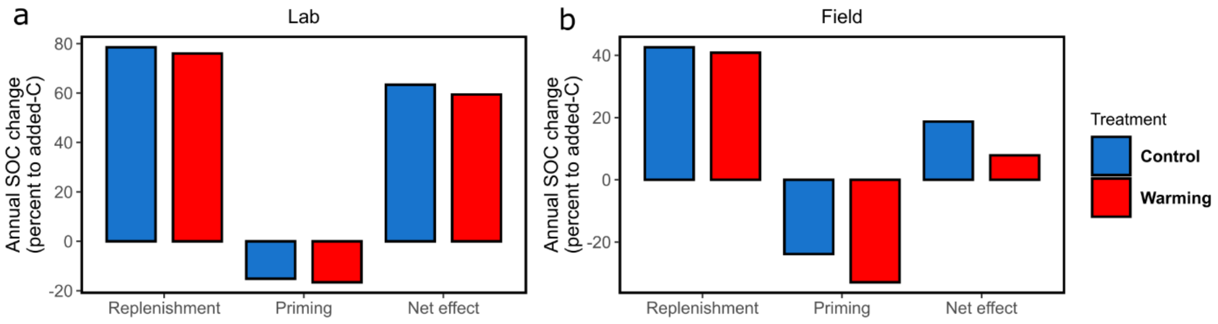

Supplementary Fig. 18 | Annual SOC change induced by replenishment and priming, and the consequent net SOC change simulated by lab-MEND and field-MEND. a. Estimation of SOC changes under lab incubation conditions. Replenishment refers to the amount of new (added) C remaining in soil C pools after microbial respiration within a year of simulation. The priming effect is the difference in C loss from native SOC between the substrate addition treatment and the control. The net effect of litter addition on SOC change is the difference between replenishment and priming. The change in SOC induced by each process was scaled to the initially added litter C amount. **b.** Estimation of SOC changes under field conditions in 2016. For the field condition, litter addition is replaced by plant carbon input. The priming effect is the difference in C loss from native SOC between the normal condition (with plant carbon input) and an assumed condition (without plant carbon input for a whole year). Source data are provided as a Source Data file.

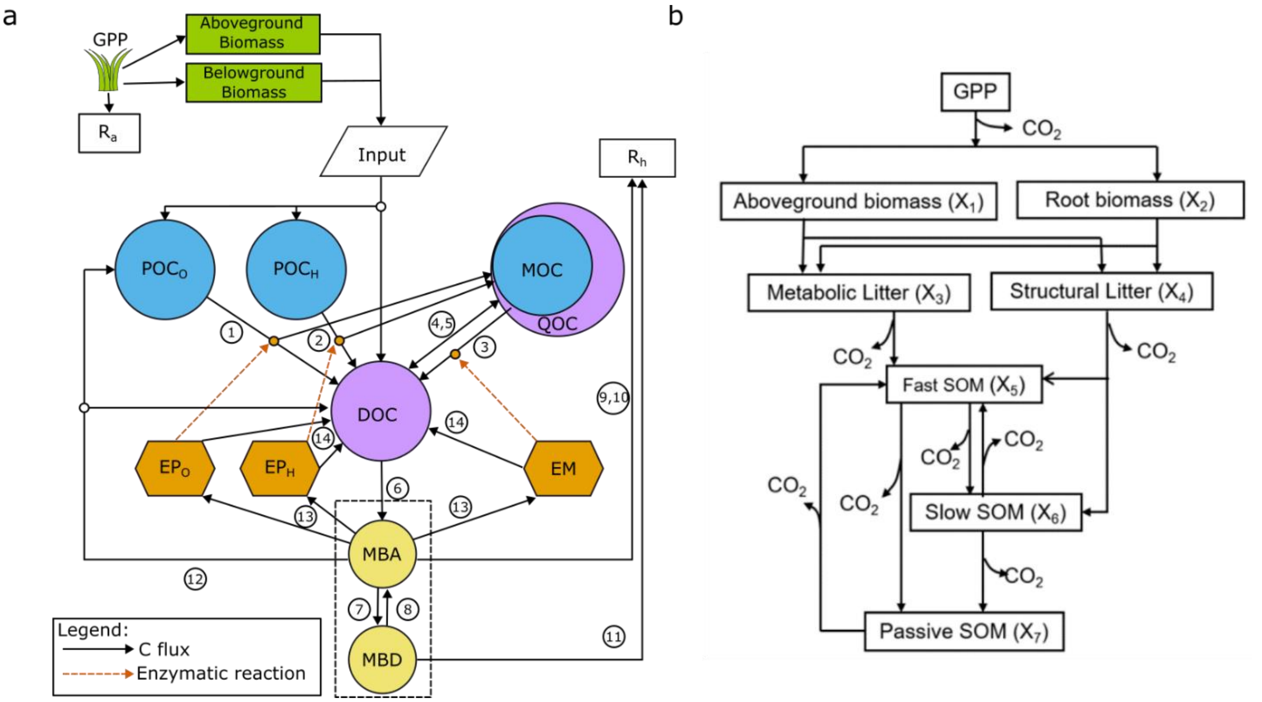

Supplementary Fig. 19 | The diagram of the Microbial-ENzyme Decomposition (MEND) model[30] and Terrestrial ECOsystem (TECO) model. a, MEND. The organic C pools include (1) particulate organic C (POC), which is divided into two components: POC_O (denoted by state variable P_O in governing equations) degraded by oxidative enzymes EP_O and POC_H (P_H degraded by hydrolytic enzymes EP_H; (2) mineral-associated organic C (MOC, *M*) degraded by enzymes EM; (3) dissolved organic C (DOC, *D*); (4) adsorbed DOC (QOC, *Q*): active MOC that adsorbs and desorbs DOC; (5) active microbial biomass (MBA, *BA*) and dormant microbial biomass (MBD, *BD*); and (6) enzyme pools EP_O, EP_H, and *EM*. External litter inputs (Inputs) can be divided into I_{PO}, I_{PH}, and I_D denoting inputs to the pools of POC_O, POC_H, and DOC, respectively. R_a represents autotrophic respiration. R_h represents heterotrophic respiration. **b**, TECO. Rh is the sum of respired CO2 during litter and soil C decomposition.

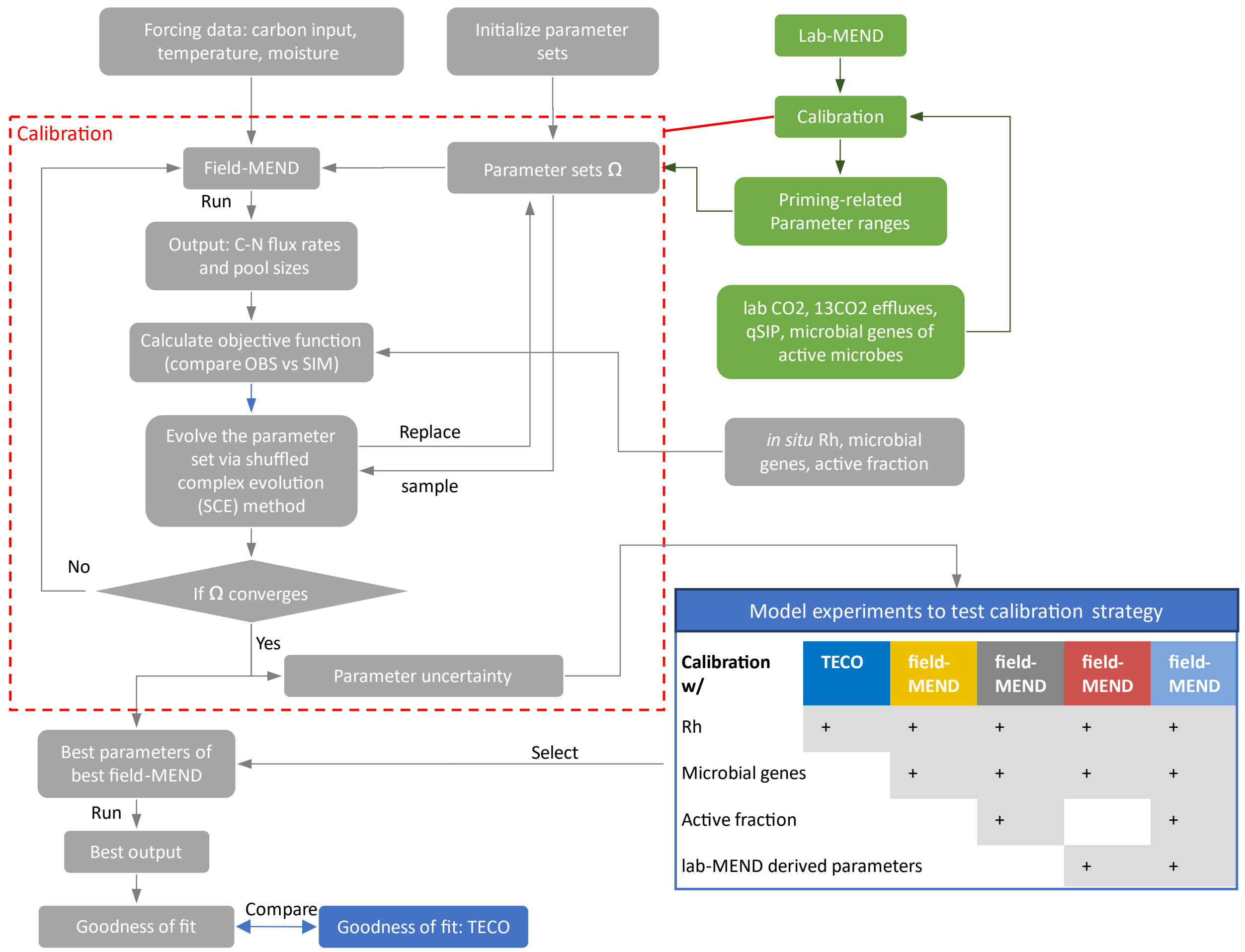

Supplementary Fig. 20 | The flowchart of model calibration and experiments with field-MEND and lab-MEND. Grey boxes mainly represent steps of field-MEND model and green boxes represent steps of lab-MEND. The arrow indicates the results of the former box serve as the input, parameters, or the model for the latter box. The field-MEND and lab-MEND shared similar calibration algorithms (the steps within the red frame) expect that field-MEND model use field warming experiment data as well as lab-MEND derived parameters to adjust the initial parameter sets used in calibration. The model experiment helps to determine the best models outside the calibration steps.

References

1. Friedrich, M. W. Stable-isotope probing of DNA: insights into the function of uncultivated microorganisms from isotopically labeled metagenomes. *Current Opinion in Biotechnology* **17**, 59-66 (2006).
2. Gallagher, E., McGuinness, L., Phelps, C., Young, L. & Kerkhof, L. 13C-carrier DNA shortens the incubation time needed to detect benzoate-utilizing denitrifying bacteria by stable-isotope probing. *Applied and Environmental Microbiology* **71**, 5192-5196 (2005).
3. Zhang, X., Han, X., Yu, W., Wang, P. & Cheng, W. Priming effects on labile and stable soil organic carbon decomposition: Pulse dynamics over two years. *PloS one* **12**, e0184978 (2017).
4. Chen, X. *et al.* Resistant soil carbon is more vulnerable to priming effect than active soil carbon. *Soil Biology and Biochemistry* **168**, 108619 (2022).
5. Conrad, R., Klose, M., Yuan, Q., Lu, Y. & Chidthaisong, A. Stable carbon isotope fractionation, carbon flux partitioning and priming effects in anoxic soils during methanogenic degradation of straw and soil organic matter. *Soil Biology and Biochemistry* **49**, 193-199 (2012).
6. Hungate, B. A. *et al.* Quantitative microbial ecology through stable isotope probing. *Appl. Environ. Microbiol.* **81**, 7570-7581 (2015).
7. Klappenbach, J. A., Dunbar, J. M. & Schmidt, T. M. rRNA operon copy number reflects ecological strategies of bacteria. *Applied and environmental microbiology* **66**, 1328-1333 (2000).
8. Tao, X. *et al.* Winter warming in Alaska accelerates lignin decomposition contributed by Proteobacteria. *Microbiome* **8**, 1-12 (2020).
9. Bugg, T. D., Ahmad, M., Hardiman, E. M. & Singh, R. The emerging role for bacteria in lignin degradation and bio-product formation. *Current opinion in biotechnology* **22**, 394-400 (2011).
10. Amore, A., Pepe, O., Ventorino, V., Aliberti, A. & Faraco, V. Cellulolytic Bacillus strains from natural habitats-a review. *Chimica Oggi/Chemistry Today* **31**, 49-52 (2013).
11. Chen, J. *et al.* A keystone microbial enzyme for nitrogen control of soil carbon storage. *Science advances* **4**, eaaq1689 (2018).
12. Klappenbach, J. A., Dunbar, J. M. & Schmidt, T. M. rRNA operon copy number reflects ecological strategies of bacteria. *Appl. Environ. Microbiol.* **66**, 1328-1333 (2000).
13. Condon, C., Liveris, D., Squires, C., Schwartz, I. & Squires, C. L. rRNA operon multiplicity in Escherichia coli and the physiological implications of rrn inactivation. *Journal of bacteriology* **177**, 4152-4156 (1995).
14. Li, J. *et al.* Predictive genomic traits for bacterial growth in culture versus actual growth in soil. *The ISME journal* **13**, 2162-2172 (2019).
15. Ning, D. *et al.* A quantitative framework reveals ecological drivers of grassland microbial community assembly in response to warming. *Nature communications* **11**, 1-12 (2020).
16. Fontaine, S., Mariotti, A. & Abbadie, L. The priming effect of organic matter: a question of microbial competition? *Soil Biology and Biochemistry* **35**, 837-843 (2003).
17. Blagodatskaya, E. & Kuzyakov, Y. Mechanisms of real and apparent priming effects and their dependence on soil microbial biomass and community structure: critical review. *Biology and Fertility of Soils* **45**, 115-131 (2008).
18. Kuzyakov, Y. Priming effects: interactions between living and dead organic matter. *Soil Biology and Biochemistry* **42**, 1363-1371 (2010).
19. Pascault, N. *et al.* Stimulation of different functional groups of bacteria by various plant residues as a driver of soil priming effect. *Ecosystems* **16**, 810-822 (2013).

20 Morrissey, E. M. *et al.* Bacterial carbon use plasticity, phylogenetic diversity and the priming of soil organic matter. *The ISME journal* **11**, 1890-1899 (2017).
21 Goldford, J. E. *et al.* Emergent simplicity in microbial community assembly. *Science* **361**, 469-474 (2018).
22 Wang, G., Post, W. M. & Mayes, M. A. Development of microbial ‐ enzyme ‐ mediated decomposition model parameters through steady ‐ state and dynamic analyses. *Ecological Applications* **23**, 255-272 (2013).
23 Jian, S. *et al.* Multi-year incubation experiments boost confidence in model projections of long-term soil carbon dynamics. *Nature communications* **11**, 1-9 (2020).
24 Greener, J. G., Kandathil, S. M., Moffat, L. & Jones, D. T. A guide to machine learning for biologists. *Nature Reviews Molecular Cell Biology* **23**, 40-55 (2022).
25 Luo, Y., Hui, D. & Zhang, D. Elevated CO2 stimulates net accumulations of carbon and nitrogen in land ecosystems: A meta ‐ analysis. *Ecology* **87**, 53-63 (2006).
26 Chen, R. *et al.* Soil C and N availability determine the priming effect: microbial N mining and stoichiometric decomposition theories. *Global change biology* **20**, 2356-2367 (2014).
27 Craine, J. M., Morrow, C. & Fierer, N. Microbial nitrogen limitation increases decomposition. *Ecology* **88**, 2105-2113 (2007).
28 Kuzyakov, Y., Friedel, J. K. & Stahr, K. Review of mechanisms and quantification of priming effects. *Soil Biol Biochem* **32**, 1485-1498 (2000).
29 Blagodatskaya, E. & Kuzyakov, Y. Mechanisms of real and apparent priming effects and their dependence on soil microbial biomass and community structure: critical review. *Biology and Fertility of Soils* **45**, 115-131 (2008). https://doi.org:10.1007/s00374-008-0334-y
30 Ying, X. in *Journal of physics: Conference series.* 022022 (IOP Publishing).
31 Wang, G. S. *et al.* Microbial dormancy improves development and experimental validation of ecosystem model. *Isme J* **9**, 226-237 (2015). https://doi.org:10.1038/ismej.2014.120
32 Wang, G. S. *et al.* Soil moisture drives microbial controls on carbon decomposition in two subtropical forests. *Soil Biol Biochem* **130**, 185-194 (2019). https://doi.org:10.1016/j.soilbio.2018.12.017
33 Wang, G. S., Post, W. M. & Mayes, M. A. Development of microbial-enzyme-mediated decomposition model parameters through steady-state and dynamic analyses. *Ecological Applications* **23**, 255-272 (2013).
