# Supplementary material for: Experimental warming accelerates positive soil priming in a temperate grassland ecosystem
Source: Nat Commun. 2024 Feb 8;15:1178. doi: 10.1038/s41467-024-45277-0 (PMC10853207; doi:10.1038/s41467-024-45277-0)
Supplement: Supplementary file 2 — Reporting Summary [file 41467_2024_45277_MOESM2_ESM.pdf]

nature portfolio

Corresponding author(s): Jizhong Zhou

Last updated by author(s): Jan 15, 2024

Reporting Summary

Nature Portfolio wishes to improve the reproducibility of the work that we publish. This form provides structure for consistency and transparency in reporting. For further information on Nature Portfolio policies, see our Editorial Policies and the Editorial Policy Checklist.

Statistics

For all statistical analyses, confirm that the following items are present in the figure legend, table legend, main text, or Methods section.

n/a	Confirmed	
☐	☒	The exact sample size (*n*) for each experimental group/condition, given as a discrete number and unit of measurement
☐	☒	A statement on whether measurements were taken from distinct samples or whether the same sample was measured repeatedly
☐	☒	The statistical test(s) used AND whether they are one- or two-sided<br>*Only common tests should be described solely by name; describe more complex techniques in the Methods section.*
☐	☒	A description of all covariates tested
☐	☒	A description of any assumptions or corrections, such as tests of normality and adjustment for multiple comparisons
☐	☒	A full description of the statistical parameters including central tendency (e.g. means) or other basic estimates (e.g. regression coefficient) AND variation (e.g. standard deviation) or associated estimates of uncertainty (e.g. confidence intervals)
☐	☒	For null hypothesis testing, the test statistic (e.g. *F*, *t*, *r*) with confidence intervals, effect sizes, degrees of freedom and *P* value noted<br>*Give P values as exact values whenever suitable.*
☒	☐	For Bayesian analysis, information on the choice of priors and Markov chain Monte Carlo settings
☒	☐	For hierarchical and complex designs, identification of the appropriate level for tests and full reporting of outcomes
☐	☒	Estimates of effect sizes (e.g. Cohen's *d*, Pearson's *r*), indicating how they were calculated

Our web collection on statistics for biologists contains articles on many of the points above.

Software and code

Policy information about availability of computer code

Data collection	All the software used in current study for data collection are commercial.<br>Feature Extraction Software (Agilent v 2.2.21) was used to grid and process the images of GeoChip to transform them into raw signal intensity.<br>Sequencing reads were undertaken with MiSeq platform(Illumina, SanDiego, CA, USA).<br>Daily GPP values were obtained from a corrected 8-day GPP product based on the MODIS GPP (MOD17A2/MOD17A2H).
Data analysis	Raw amplicon data were processed and analyzed using the Galaxy-based pipeline published and developed by the Institute for Environmental Genomics, University of Oklahoma accessible by public at http://ieg3.rccc.ou.edu:8081/. ASVs were clustered by using DADA2 R package (version.1.12) . Geochip raw data were processed and analyzed in the Microarray Data Manager in our website (https://www.ou.edu/ieg/tools/data-analysis-pipeline). Statistical analyses were performed in R version 4.2.2 (www.R-project.org) and package "vegan" (version 2.4.6), package “entropart” (version version 1.6-13), “iCAMP” (version version 1.6.1), package "glmulti" (version 1.0.8), package "ropls" (version 1.34.0), package "lmPerm" (version.2.1.0), package "stats" (version 3.5.2), package "lme4" (version 1.1-35.1) and package "emmeans" (version 1.9.0) . Detailed information is provided in the text. The codes for modeling analysis are performed in FORTRAN and are available in Zenodo with the DOI identifier at https://doi.org/10.5281/zenodo.10498280. We used the Shuffled Complex Evolution (SCE) algorithm to determine model parameters. We also applied the Critical Objective Function Index (COFI) method to quantity parameter uncertainties.

For manuscripts utilizing custom algorithms or software that are central to the research but not yet described in published literature, software must be made available to editors and reviewers. We strongly encourage code deposition in a community repository (e.g. GitHub). See the Nature Portfolio guidelines for submitting code & software for further information.

Data

Policy information about availability of data

All manuscripts must include a data availability statement. This statement should provide the following information, where applicable:

- Accession codes, unique identifiers, or web links for publicly available datasets
- A description of any restrictions on data availability
- For clinical datasets or third party data, please ensure that the statement adheres to our policy

Raw sequences of 16S rRNA gene amplicons after the 7-day incubation with straw are available in the NCBI SRA database (http://www.ncbi.nlm.nih.gov/sra) under accession number PRJNA595391 (https://www.ncbi.nlm.nih.gov/bioproject/?term=PRJNA595391). Raw sequences of 16S rRNA gene amplicons for yearly warming field sites are available in the NCBI SRA database (http://www.ncbi.nlm.nih.gov/sra) under accession number PRJNA331185 (https://www.ncbi.nlm.nih.gov/bioproject/?term=PRJNA331185). GeoChip raw and normalized signal intensities are deposited in the BioStudies under accession number E-MTAB-13326 (https://www.ebi.ac.uk/biostudies/arrayexpress/studies/E-MTAB-13326). All other relevant data are available in Supplementary Information. MEND model codes are available in Zenodo with the DOI identifier at https://doi.org/10.5281/zenodo.10498280. Source data are provided with this paper.

Research involving human participants, their data, or biological material

Policy information about studies with human participants or human data. See also policy information about sex, gender (identity/presentation), and sexual orientation and race, ethnicity and racism.

Reporting on sex and gender	N/A
Reporting on race, ethnicity, or other socially relevant groupings	N/A
Population characteristics	N/A
Recruitment	N/A
Ethics oversight	N/A

Note that full information on the approval of the study protocol must also be provided in the manuscript.

Field-specific reporting

Please select the one below that is the best fit for your research. If you are not sure, read the appropriate sections before making your selection.

☐ Life sciences ☐ Behavioural & social sciences ☒ Ecological, evolutionary & environmental sciences

For a reference copy of the document with all sections, see nature.com/documents/nr-reporting-summary-flat.pdf

Ecological, evolutionary & environmental sciences study design

All studies must disclose on these points even when the disclosure is negative.

Study description	Our field experiment was conducted at the Kessler Atmospheric and Ecological Field Station (KAEFS) in McClain County, Oklahoma, USA (34°59' N, 97°31' W), initiated in July 2009. The design employed was a block structure, focusing solely on warming (+3 °C above ambient) as the treatment factor, without any nested or hierarchical designs involved. The experiment was divided into two groups: the warming treatment and the control group. Each group consisted of four replicates, distributed across four different blocks, resulting in a total of eight subplots.
Research sample	In our study, we focus on the priming effect, active microbial community, and their functions in response to warming in the temperate grassland ecosystem. Therefore, we collected top soil samples in 2016, which were then specifically used for studying the priming effect, the active microbial community. The corresponding microbial-based mechanisms were further incorporated into our ecosystem modeling. At same time, in order to confirm our active microbial community results, we also collected the in situ field microbial community data during 2010 to 2016 from our previous study (Guo, et al., Nature Communications, 2020). In conclusion, this approach allows us to directly link long-term field observations with detailed microbial analyses, offering a comprehensive view of the temperate grassland's response to climate change.
Sampling strategy	In this study, we collected 8 surface soil samples at peak plant biomass (September 2016), including four from warmed plots and four from control plots. Each sample consisted of three composited soil cores (2.5 cm diameter x 15 cm deep) to ensure enough material for comprehensive analyses. The choice of eight samples, constrained by the availability of four warming and four control plots, was specifically aligned with our focus on the warming effect. This sample size has been proven effective in our previous studies (Guo, et al., Nature Climate Changes, 2018; Guo, et al., Nature Communications, 2020; Wu et al., Nature Microbiology, 2022), which found significant warming impacts on total soil microbial community structures, diversities, and respiration, demonstrating the sample size's statistical power to capture microbial responses to warming.
Data collection	Soil temperature was measured every 15min at depth of 7.5, 20, 45, and 75cm in the center of each plot using constantan-copper

thermo couples wired to a Campbell Scientific CR10x data logger (Campbell Scientific). The data were recorded and backed up by Zheng Shi in computers periodically. Volumetric soil water content (%V) was measured using a portable time domain relectometer (Soil Moisture Lquipment Corp.) once or twice a month, and recorded by pen and paper. Ecosystem carbon (C) fluxes were measured once or twice a month between 10:00 and 15:00 (CDT). Net ecosystem exchange and ecosystem respiration were measured using an LI-6400 portable photosynthesis system (LI-COR) attached to a transparent chamber (0.5m x 0.5m x 0.7m), which covered all of the vegetation within the aluminium frames. The LI-6400 system had storage to record the data. Soil total respiration and heterotrophic respiration were measured using a LI-8100A soil flux system attached to a soil CO2 flux chamber (LI-COR). The LI-8100A system had storage to record the data. Meanwhile, a manual record (by pen and paper) was also kept. Soil water content and ecosystem carbon fluxes were measured monthly or biweekly by the field team, including Linwei Wu, Ya Zhang, Xue Guo, Daliang Ning, Liyou Wu, Xishu Zhou, Jiajie Feng, Xuanyu Tao, Jjaliang Kuang, and Zheng Shi. Soil and plant straw chemical properties were analyzed in Forage Analytical Lab at Oklahoma State University. The results were sent to Siyang Jian and Xuanyu Tao through emails and stored in computers. All soil sample collection from the experiment site was performed by authors XG, JF, XT, XZ, and the lab technicians. Three soil cores (2.5 cm diameter x 15 cm deep) were collected in each field plots using a soil sampler tube and composited to have enough samples for soil chemistry, microbiology and molecular biology analyses. Soil DNA extraction and PCR were performed by XG, JF, XT and XZ in the University of Oklahoma. GeoChip hybridization and MiSeq sequencing were performed by XT and JF using NimbleGen MS200 scanner and Illumina MiSeq platform. DOM analysis of the soil samples from 2016 were performed by JW using Fourier transform ion cyclotron resonance mass spectrometry(FT-ICR MS, Bruker Daltonics, Billerica, MA,USA). BIOLOG analysis was performed by XG using Biolog EcoPlates (Biolog Inc., Hayward, CA, USA).

Timing and spatial scale

In this study, We collected eight soil samples in September, 2016. Specifically, a total of Eight top soil samples (0–15 cm) was collected from 4 warmed and 4 control plots (September 2016) in this long-term warming experiment site (34 59′ N, 97 31′W). The data of ecosystem C fluxes, soil respirations and plant biomass were also collected monthly.

Data exclusions

There were no data exclusions.

Reproducibility

Each treatment has 4 replicate plots within the same site. 16S rRNA gene amplicons were sequenced by our in-home MiSeq platform (Illumina, SanDiego, CA, USA) using a 500-cycle v2 MiSeq reagent cartridge (Illumina). GeoChip 5.0 was used for all active community samples to analyze functional structure of the active microbial communities in warming and control samples. Statistical analyses of amplicon sequencing data and GeoChip data showed consistent results.

Randomization

Treatments were set up in a randomized block design.

Blinding

All samples taken were labeled with a single number to track samples during lab processing, but included no information as to the treatment from which it originated.

Did the study involve field work? ☒ Yes ☐ No

Field work, collection and transport

Field conditions

This experimental site was conducted in an old-field tallgrass prairie abandoned from cropping 40 years ago with light grazing until 2008. Ambrosia trifida, Solanum carolinense and Euphorbia dentate belonging to C3 forbs, and Tridens flavus, Sporobolus compositus and Sorghum halapense belonging to C4 grasses are dominant in the site. Annual mean temperature is 16.3 °C and annual precipitation is 914 mm. The soil type of this site is Mollisols with 51% of sand, 35% of silt and 13% of clay, which is a well-drained soil that is formed in loamy sediment on flood plains. The soil has a water holding capacity with 37%, neutral pH and 1.2 g cm-3 bulk density with around 1.9% total organic matter and 0.1% total nitrogen (N).

Location

The experimental site is located at the Kessler Atmospheric and Ecological Field Station (KAEFS) in the US Great Plains in McClain County, Oklahoma (34 59′ N, 97 31′W).

Access & import/export

The property on which the field experiment was built belongs to the University of Oklahoma. The acting director of the site is Meghan Bomgraars (mbomgaars@ou.edu, 405-325-5202). The authors have full access to the field site to conduct research. All the research activities conducted on site complies to national and local laws and regulations, and rules imposed by the University of Oklahoma in terms of ecological conservation and work safety.

Disturbance

Infrared heaters may disturb the grassland ecosystem. To minimize these disturbances, 'dummy' heaters were used in this study.

Reporting for specific materials, systems and methods

We require information from authors about some types of materials, experimental systems and methods used in many studies. Here, indicate whether each material, system or method listed is relevant to your study. If you are not sure if a list item applies to your research, read the appropriate section before selecting a response.

Materials & experimental systems

n/a	Involved in the study
☒	☐ Antibodies
☒	☐ Eukaryotic cell lines
☒	☐ Palaeontology and archaeology
☒	☐ Animals and other organisms
☒	☐ Clinical data
☒	☐ Dual use research of concern
☒	☐ Plants

Methods

n/a	Involved in the study
☒	☐ ChIP-seq
☒	☐ Flow cytometry
☒	☐ MRI-based neuroimaging
